# Highly Sensitive and Selective Colorimetric and Off-On Fluorescent Reversible Chemosensors for Al^3+^ Based on the Rhodamine Fluorophore

**DOI:** 10.3390/s150409097

**Published:** 2015-04-17

**Authors:** Naveen Mergu, Ashok Kumar Singh, Vinod Kumar Gupta

**Affiliations:** 1 Department of Chemistry, Indian Institute of Technology Roorkee, Roorkee 247 667, India; E-Mails: mergu.naveen@gmail.com (N.M.); akscyfcy@gmail.com (A.K.S.); 2 Center for Environment and Water, The research Institute, King Fahd University of Petroleum & Minerals, Dhahran 31261, Saudi Arabia; 3 Department of Applied Chemistry, University of Johannesburg, Johannesburg 17011, South Africa

**Keywords:** chemosensor, rhodamine, colorimetric, fluorescence, naked-eye detection

## Abstract

A series of rhodamine derivatives **L1**–**L3** have been prepared and characterized by IR, ^1^H-NMR, ^13^C-NMR and ESI-MS. These compounds exhibited selective and sensitive “turn-on” fluorescent and colorimetric responses to Al^3+^ in methanol. Upon the addition of Al(III), the spiro ring was opened and a metal-probe complex was formed in a 1:1 stoichiometry, as was further confirmed by ESI-MS spectroscopy. The chemo-dosimeters **L1**–**L3** exhibited good binding constants and low detection limits towards Al(III). We also successfully demonstrate the reversibility of the metal to ligand complexation (opened ring to spirolactam ring).

## 1. Introduction

Aluminum is the third most abundant element in the Earth's crust and second most widely used metal after iron for the manufacture of electrical equipment, automobiles, building construction, water purification, clinical drugs [1] and packaging materials [2], *etc*. Recent studies warn that the deposition of aluminum in bone and the nervous system of the human body can cause neurotoxicity in high dosage [3]. Unregulated amounts of aluminum in the human body may lead to Parkinson’s [4], Alzheimer’s [5,6] and dialysis [7] diseases. In plants, higher concentration of aluminum may affect the growth of root [8] and seed [9].

Thus, the monitoring of aluminum at the sub-micromolar level for biological, clinical and environmental purposes is highly desirable and indispensable. Various analytical techniques, including ion selective electrodes [10,11], colorimetric sensors [12,13], voltammetry [14], atomic absorption and emission spectrometry [15,16], chromatography [17] and inductively coupled plasma mass spectroscopy [18], *etc.*, have been used to determine the presence of metal ions in different samples.

Among the various detection methods, the fluorescence sensing technique [19,20,21,22,23,24,25,26,27] has become the most useful and popular in clinical, biology and environmental chemistry due to its non-destructive nature, high selectivity and sensitivity, real-time response and possible naked eye detection. The poor coordination ability of Al^3+^ compared to transition metal ions [28] is the major reason for the lesser development of Al^3+^ chemosensors. The use of the rhodamine moiety is a reliable way to construct “off-on” fluorescent probes because the sensing mechanism of rhodamine-derived probes is based on the structure change from their spirocyclic form (fluorescence “off”) to a ring-opened amide form (fluorescence “on”) induced by a specific chemical species such as a metal ion at room temperature [29,30].

**Scheme 1:**
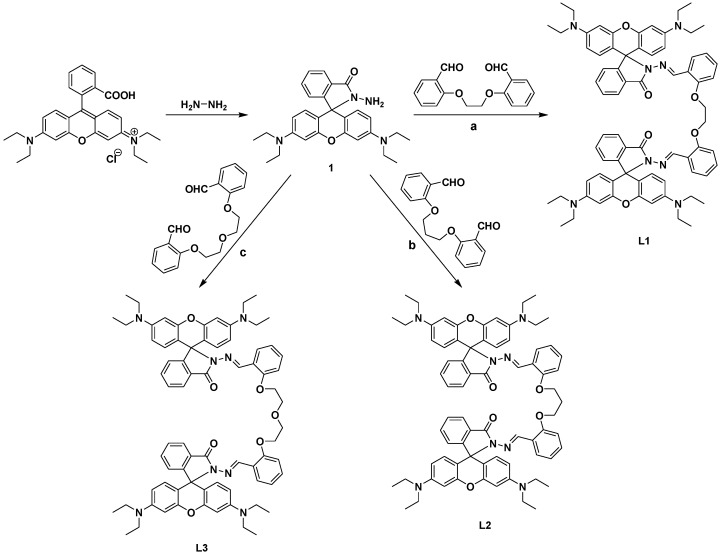
Synthetic Pathways to **L1**–**L3**.

Rhodamine derivatives are used widely as a fluorescent signal transducers due to their excellent photophysical properties such as long absorption and emission wavelengths, large absorption coefficients and high fluorescence quantum yields. In fact, fluorophores having a long emission wavelength (~550 nm) are often chosen to serve as a best signal transducers to avoid the influence of background fluorescence (below 500 nm) [31,32]. Recently, several rhodamine-based fluorescent chemosensors have been developed for different chemical species [33,34,35]. Herein, we report the synthesis of a series of rhodamine-derived chemosensors **L1**–**L3** using the synthetic route outlined in Scheme 1. First, rhodamine B hydrazide (compound **1**) was obtained from rhodamine B by treating it with hydrazine hydrate. Upon further refluxing with dialdehydes (a, b and c) compound **1** yielded the sensors **L1**, **L2** and **L3**, which show a reversible, selective and sensitive fluorescence enhancement response to Al(III) in alcoholic media.

## 2. Results and Discussion

The selectivity, sensitivity and binding mode of chemosensors toward Al^3+^ were examined through absorption, emission, ESI-MS, electrochemical (DPV) and ^1^H-NMR experiments.

### 2.1. Absorption Spectroscopic Studies

**Figure 1:**
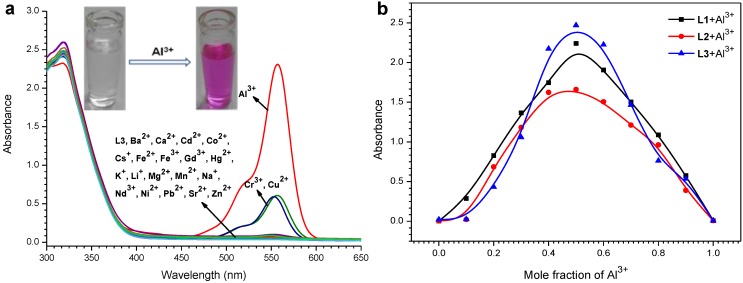
(**a**) Absorbance spectra of **L3** (50 µM) in presence of various metal ions (50 µM) in MeOH−DMSO (99:1 v/v). Inset: Visual color change of probe upon addition of Al^3+^; (**b**) Job’s plot for **L1**–**L3** with Al^3+^, absorbance intensity at 557 nm was plotted as a function of the molar ratio.

The binding capacity of the probes (50 µM) toward various metal ions (50 µM) such as Al^3+^, Ba^2+^, Ca^2+^, Cd^2+^, Co^2+^, Cr^3+^, Cs^+^, Cu^2+^, Fe^2+^, Fe^3+^, Gd^3+^, Hg^2+^, K^+^, Li^+^, Mg^2+^, Mn^2+^, Na^+^, Nd^3+^, Ni^2+^, Pb^2+^, Sr^2+^ and Zn^2+^ was measured by UV-vis absorption studies. For example, the UV-vis spectrum of **L3** exhibited a main absorption band in the 300–400 nm range. When aluminum ion was added to the probe, a strong absorption band at ~557 nm with a shoulder at ~519 nm appeared (Figure 1a). A weak absorption peak was observed with Cr^3+^ and Cu^2+^, while no peak appeared with the other metal ions even when in excess.

The observable naked eye detection of the development of a magenta color in the probe (Figure 1a, inset) upon Al(III) addition involves a metal-induced delactonization of rhodamine. On complexation, the primary spirolactam form of the probe is converted into its ring opened amide conformation [36]. The plot of absorbance of **L3** at 557 nm as a function of mole fraction of added Al^3+^ metal ion (Jobs plot) reveals that these probes bind to the metal ion in 1:1 stoichiometry (Figure 1b).

### 2.2. Fluorescence Emission Studies

**Figure 2:**
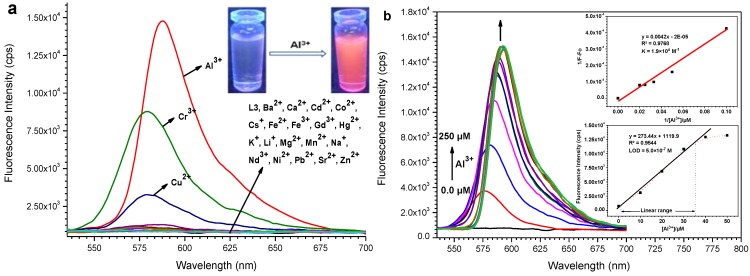
(**a**) Fluorescence spectra (*λ*_ex_ = 520 nm) of **L3** (50 µM) in presence of various metal ions (50 µM) in MeOH−DMSO (99:1 v/v). Inset: Visual color change of probe upon addition of Al^3+^; (**b**) The fluorescence emission spectral pattern of **L3** in the presence of increasing concentrations of Al^3+^ (0, 10, 20, 30, 40, 50, 75, 100, 125, 150, 175, 200, 225, 250 µM). Inset: Linear regression plot of fluorescence intensity change 1/(*F*-*F*_0_) as a function of concentration 1/[Al^3+^] (top), fluorescence enhancement change as a function of concentration of Al(III) added (bottom).

The fluorescence spectral pattern of **L3** when excited at 520 nm in the presence of various metal ions (Figure 2a) exposed that the non-fluorescent (OFF) behaviour becomes highly fluorescent (ON). Under a UV lamp, a spectacular color change of the probe solution from colorless to fluorescent pink occurred upon addition of Al^3+^, which could simply be detected by the naked-eye (Figure 2a, inset). This involves a delactonization process of the spirocyclic (non-fluorescent) form of rhodamine to its ring opened (highly fluorescent) form which is induced by metal ion coordination. The amount of chelation-enhanced fluorescence depends on nature of the ionophores and interacting metal ions. Among all the metal ions examined, these probes demonstrated high fluorescence enhancement at *λ*_em_ = ~587 nm in the presence of Al(III) ion.

**Figure 3:**
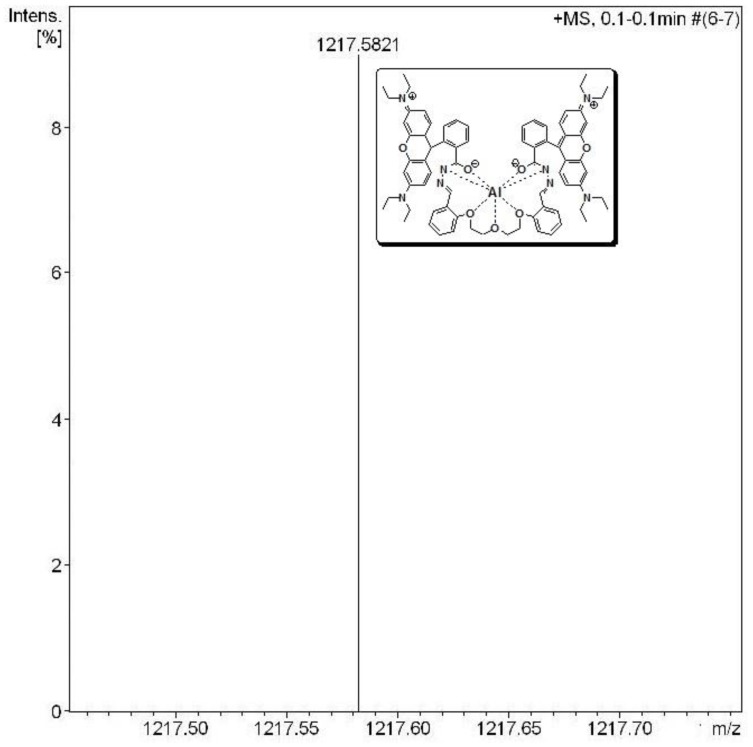
ESI-MS spectrum of **L3** upon addition of AlCl_3_·6H_2_O (1.0 eqiuv.) in MeOH.

The fluorescence emission spectral pattern of **L1**–**L3** (50 µM) upon addition of increasing concentrations (0, 10, 20, 30, 40, 50, 75, 100, 125, 150, 175, 200, 225, 250 µM) of Al^3+^ ion changed, with a new emission band peak appearing at ~587 nm that increased in intensity with increasing Al^3+^ concentration (Figure 2b and Figure S3). The complex stability constants (*K*) calculated through the Benesi-Hildebrand method for Al(III) with **L1**–**L3** were found to be 5.7 × 10^3^ M^−1^, 1.6 × 10^4^ M^−1^ and 1.9 × 10^4^ M^−1^, respectively (Figure 2b and Figure S3, inset). The observable development of a fluorescent pink color in these probes is due to the formation of a highly delocalized π-conjugated probe system. The fluorescence enhancement is linearly proportional to Al^3+^ concentration from 2.1 × 10^−5^ M to 5.0 × 10^−5^ M (for **L1**), 1.0 × 10^−6^ M to 5.0 × 10^−5^ M (for **L2**) and 1.0 × 10^−6^ M to 3.5 × 10^−5^ M (for **L3**), and the detection limit of Al(III) was estimated based on the fluorescence titration profile as 6.0 × 10^−7^ M (for **L1**), 5.8 × 10^−7^ M (for **L2**) and 5.0×10^−7^ M (for **L3**) based on S/N = 3 (Figure 2b and Figure S3, inset). Moreover, to determine the stoichiometry of the probe-Al^3+^ complexes, the continuous variation (Job’s) method was used (Figure S4). As expected, the results suggested a 1:1 stoichiometry Al^3+^ to probe for the complexes, which was further proved by ESI-MS analysis (Figure 3 and Figure S5). Observed mass peaks at *m*/*z* 1173.5524, 1187.5739 and 1217.5821 corresponding to [**L1** + Al]^+^, [**L2** + Al]^+^ and [**L3** + Al]^+^, respectively, were observed, which are solid proof for the formation of a 1:1 complex.

**Figure 4:**
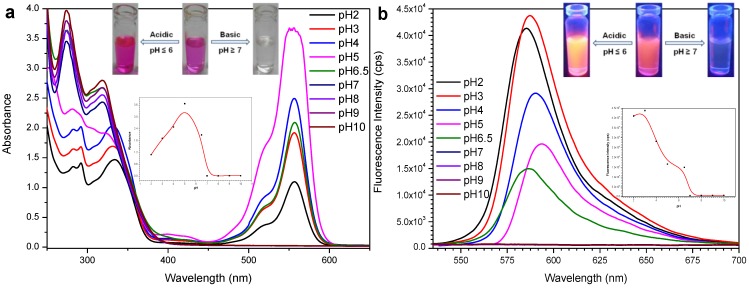
UV-vis absorbance (**a**) and fluorescence emission (**b**) spectral changes of **L3** with Al^3+^ as a function of pH. Inset: Color changes of probe + Al^3+^ in different pH media under a normal (**a**) and UV (**b**) lamp (top), absorbance (**a**, at 557 nm) and emission (**b**, at 587 nm) intensities of **L3** in the presence of Al^3+^ with pH variation (bottom).

Normally, the spiro rings of rhodamine and its derivatives are disturbed in acidic media and then show the absorbance and fluorescence characteristics of rhodamine. The absorbance and fluorescence responses of probe **L3** in the presence of Al(III) in different pH values were estimated (Figure 4). The pH was adjusted by using dilute hydrochloric acid and sodium hydroxide. Initially, the metal solutions were prepared while adjusting the pH, then the sensor was further added into these solutions under vigorous stirring for 1 min. The probe-Al(III) absorbance gradually increased from pH 2 to 5 and reached a *λ*_max_ at pH 5. From pH 5 to 6.5 (neutral), the absorbance maxima moved downward. A small fluorescence enhancement accompanied by a red shift was observed with a pH change from 2 to 3, and a rapid fluorescence quenching accompanied by a red shift started while changing the pH from 3 to 5. Again, a quenching was observed with a blue shift from pH 5 to 6.5. A rigid structure is formed when interacting with H^+^ ions from acids and this can lead to a red shift. The absorbance (at 557 nm) and fluorescence emission (at 587 nm) bands of the probe-Al(III) complex disappeared under basic conditions (pH ≥ 7). The same spectral changes were observed for probe **L3** alone under various pH conditions. The chemosensor **L3** in the presence of Al(III) exhibited intense color changes in the different pH media, which could easily be detected by the naked-eye (Figure 4, inset).

**Figure 5:**
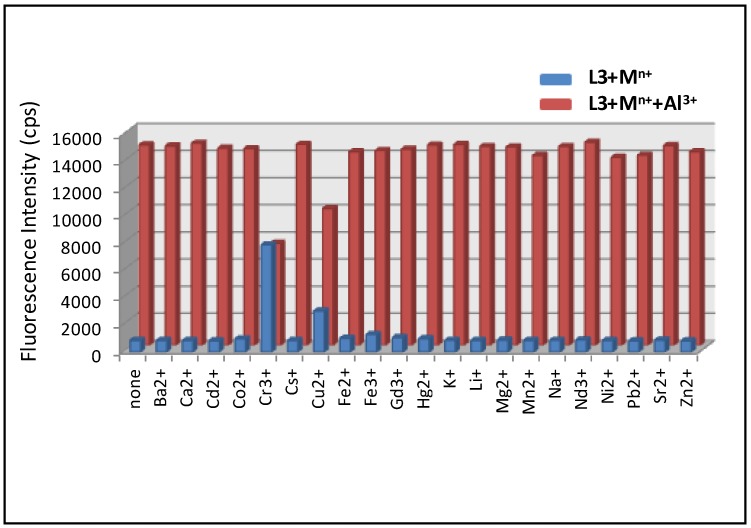
Competitive selectivity of probe **L3** toward various metal ions (1.0 equiv.) in the absence (blue bars) and presence (red bars) of Al^3+^ (1.0 equiv.) with an excitation of 520 nm.

To verify the selectivity of these probes towards Al(III) over other competitive metal ions, the emission intensity changes of **L3** (50 µM) upon addition of other metal ions (50 µM) along with Al(III) were evaluated (Figure 5). The results revealed that Al(III)-induced fluorescence response was affected by the presence of Cr(III) and Cu(II), and uninfluenced in the presence of other potentially interfering ions. This experiment establishes the significant high selectivity of these probes towards Al(III) over other competitive metal ions (except Cr^3+^ and Cu^2+^).

**Figure 6:**
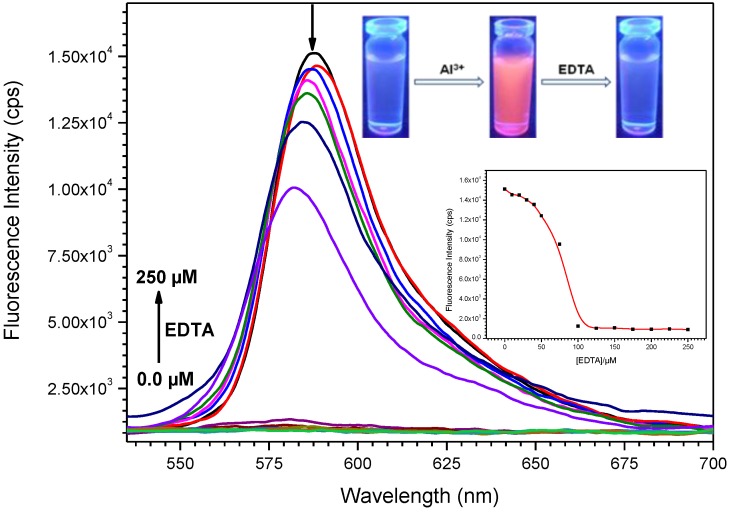
The variation in fluorescence emission spectra of **L3** + Al^3+^ upon addition of EDTA (0, 10, 20, 30, 40, 50, 75, 100, 125, 150, 175, 200, 225, 250 µM). Inset: Color changes of probe + Al^3+^ upon addition of EDTA (1.0 equiv.) (**top**), fluorescence spectral changes at 587 nm as a function of the amount of EDTA (**bottom**).

**Scheme 2:**
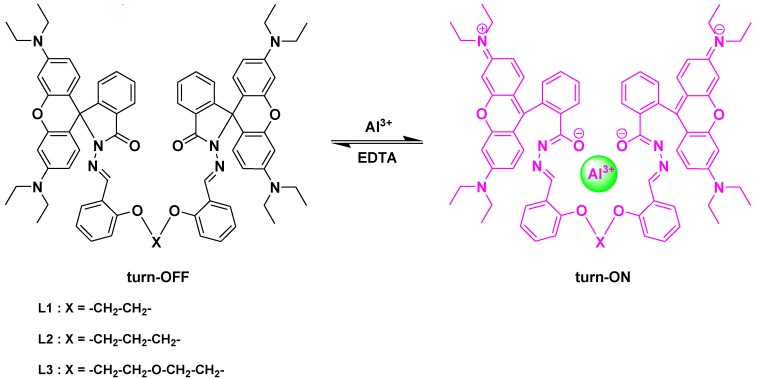
Proposed binding mechanism of Al(III) with probes in the presence and absence of EDTA.

To examine the reversibility of the probe complexation with Al(III) ion, EDTA titration experiments were performed. Upon addition of EDTA to the solution containing probe **L3** and Al(III) the fluorescence diminished significantly, whereas readdition of Al(III) ion could recover the fluorescence emission signal (Figure 6). All these results provide experimental evidence to support the reversibility of the spiro ring-opening and closing mechanism. The proposed binding mechanism of probes **L1**–**L3** with Al(III) in the presence and absence of EDTA was shown in Scheme 2.

Both the absorbance and fluorescence emission results indicate that probes show a good selectivity and sensitivity toward Al(III) over other metal ions (except Cr^3+^ and Cu^2+^). Absorbance, Emission enhancement factor and its related quantum yields of (**L1**–**L3**) in the absence and presence of Al(III) are collected in Table 1.

**Table 1:** Absorbance and emission enhancement factor, and corresponding quantum yields of **L1**–**L3** in the presence of Al(III).

System	Absorbance EF (*I*/Io*)	Emission EF (*F*/Fo*)	Quantum yield (*Ф*)
**L1**	1	1	<0.001
**L1** + Al^3+^	616	14	0.11
**L2**	1	1	<0.001
**L2** + Al^3+^	469	17	0.059
**L3**	1	1	<0.001
**L3** + Al^3+^	577	17	0.054

* Io = Absorbance of probe at neutral pH at 557 nm, *Fo* = Emission intensity of probe at neutral pH at 587 nm.

### 2.3. Electrochemical Measurements

**Figure 7:**
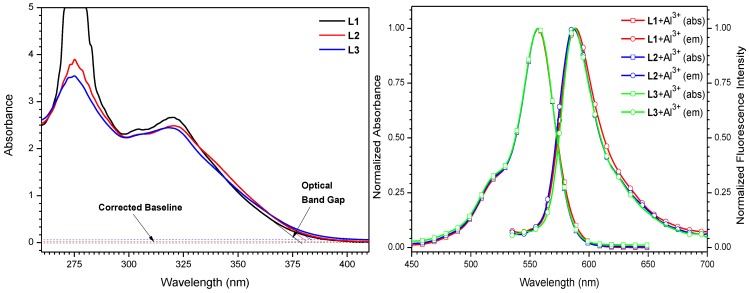
(**a**) Absorption spectra and optical band gaps of probes **L1**–**L3**; (**b**) UV-vis absorption and fluorescence emission spectra of ligands and the corresponding Al^3+^ addition products in MeOH−DMSO (99:1 v/v).

The wavelength regarding to the band gap energy for probes is obtained from the cross point of the absorption onset line and the corrected baseline (Figure 7a) [37]. The corresponding wavelengths are 379, 381 and 383 nm, and are equal to energy band gaps of 3.27, 3.25 and 3.24 eV for **L1**, **L2** and **L3** respectively.

The corresponding wavelength to band gap energy for probes with Al(III) can also be calculated from the cross point of absorption and emission onset lines (Figure 7b). The corresponding wavelengths are 573, 572 and 574 nm, which are equal to energy band gaps of 2.16, 2.17 and 2.16 eV for **L1** + Al(III), **L2** + Al(III) and **L3** + Al(III), respectively.

**Figure 8:**
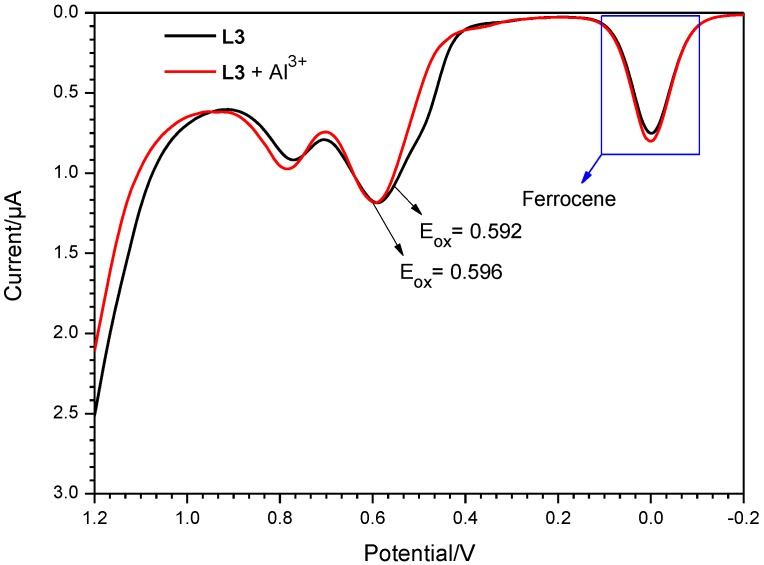
Differential pulse voltammograms recorded for **L3**, and the corresponding Al^3+^ addition product in MeOH − DMSO (99:1 v/v).

Figure 8 and S9 shows the current-voltage curve obtained for probes **L1**–**L3** in the absence and presence of Al(III) in Differential Pulse Voltammetric experiments. As the results show, **L1**, **L2** and **L3** alone show oxidation potentials of 0.584, 0.572 and 0.592 V which are equal to E_HOMO_ = −5.38, −5.37 and −5.39 eV, respectively. The corresponding oxidation potentials for **L1**–**L3** in the presence of Al(III) ions are 0.588, 0.592 and 0.596 V, which are equal to E_HOMO_ = −5.39, −5.39 and −5.40 eV, respectively. By addition of Al(III) ion, changes occurred in the oxidation potentials of the probes due to the decrease in the electron releasing nature of the probe-Al^3+^ complexes. LUMO energy levels were estimated as −2.11, −2.12, −2.15, −3.23, −3.22 and −3.24, respectively, from the HOMO and band gap energies.

**Figure 9:**
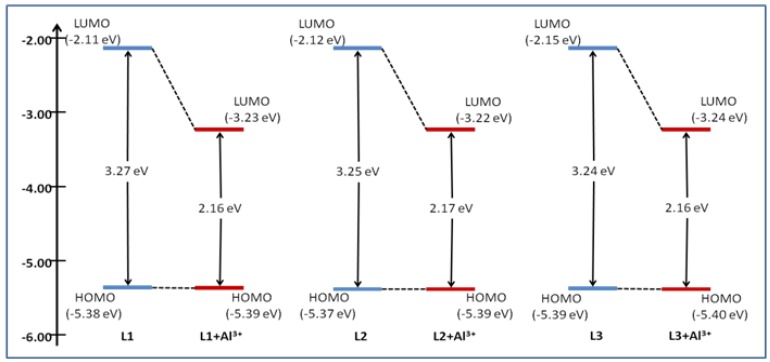
Energy level diagram of the probes and the corresponding Al^3+^ addition products.

This experiment proves an increase in oxidation potential and a decrease in band gap due to strong interactions between the probes **L1**–**L3** and aluminum ion. Figure 9 shows the energy diagram with the HOMO/LUMO levels of probes alone and in the presence of Al(III).

### 2.4. ^1^H-NMR Titration

**Figure 10:**
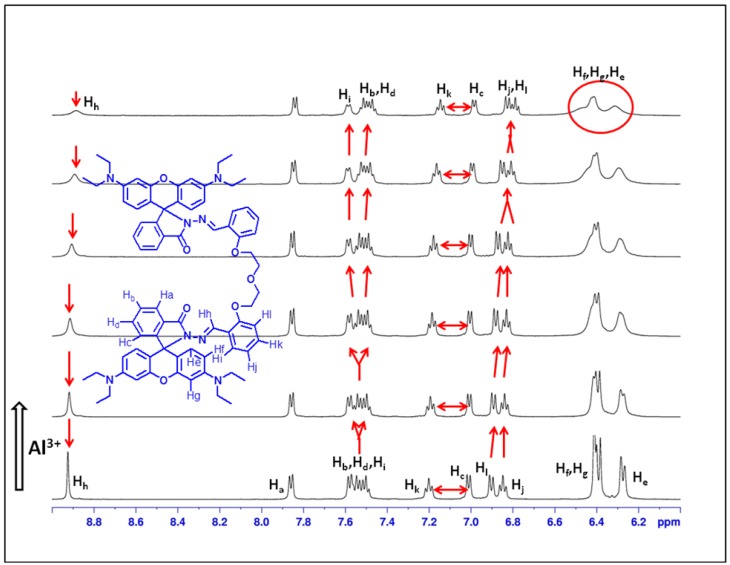
^1^H-NMR titration of **L3** with Al^3+^ in DMSO-d6 + CD_3_OD.

To better usnderstand the interaction between the probe **L3** and Al^3+^, proton NMR titration experiments were performed in the presence of various amounts of Al^3+^ in a DMSO-d_6_ + CD_3_OD solvent mixture (Figure 10). Upon complexation with Al^3+^ ion, the signal of the imine proton (H_h_) of the Schiff base moiety at 8.82 ppm steadily disappeared. Similarly the signals of the aryl-protons H_e_, H_f_ and H_g_ of the rhodamine moiety came together then to give a typical complex signal (see Supplementary Information for more details).

**Figure 11:**
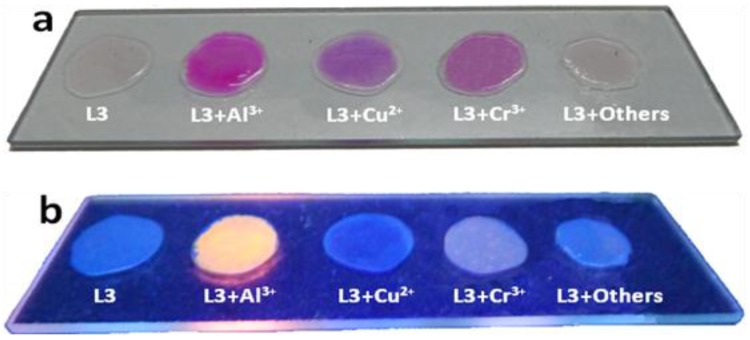
Photographs of ligand doped PVC polymeric thin films under visible (**a**) and fluorescent light (**b**).

To investigate the practical application of sensing probes **L1**–**L3**, polymeric thin films were prepared [38]. Polyvinyl chloride (PVC, 100 mg), bis(2-ethylhexyl)sebacate (as plasticizer, 200 mg) and probe were dissolved in THF (5 mL). The homogeneous mixture obtained after completion of dissolution of all ingredients was concentrated by evaporation of the THF at room temperature. This homogeneous mixture was poured onto a clean glass surface. The solvent was allowed to evaporate to give a non-fluorescent polymeric membrane sensor that was used for Al^3+^ detection. A solution containing Al^3+^ in methanol (1 mM) was sprayed onto the film, and upon evaporation of the solvent a strong fluorescent image appeared on the Al^3+^ exposed regions (Figure 11). Both Cu^2+^ and Cr^3+^ ions can also be detected colorimetrically by using this membrane, whereas the Cr^3+^ complex showed a weak fluorescence and the complex of Cu^2+^ with the **L3** chemosensor is fluorescence inactive. Thus, it could be used for simultaneous detection of Al^3+^ and Cu^2+^.

## 3. Experimental Section

### 3.1. Reagents and Apparatus

Rhodamine B, metal salts and other commercially available chemicals were purchased from Merck (Mumbai, India) and Aldrich (Bangalore, India) and used without further purification. The melting points were recorded on an OptiMelt Automated melting point system (SRS, CA, USA). The IR spectra were measured on a PerkinElmer FT-IR spectrometer (CA, USA) in the range 4000–400 cm^−1^ with KBr. The NMR spectra were recorded by using a Bruker 500 MHz instrument (MA, USA), using TMS as an internal standard, CDCl_3_, DMSO-d_6_, and CD_3_OD are taken as solvents. The mass spectra were obtained on a Bruker-micrOTOF II (MA, USA). The UV-vis absorption spectra were recorded on a Shimadzu UV-2450 spectrophotometer (Kyoto, Japan) and the Fluorescent spectra were recorded by using Horiba FluoroMax-4 spectrofluorophotometer (Kyoto, Japan). Differential Pulse Voltammetric experiments were performed using a CHI760E electrochemical workstation (Austin, TX, USA) with a conventional three-electrode configuration consisting of a glassy carbon working electrode, a platinum wire counter electrode, and a calomel reference electrode.

### 3.2. Synthesis and Characterization

Chemosensors **L1**–**L3** were prepared by following the literature method [39] and the structures were characterised by FT-IR, ^1^H-NMR, ^13^C-NMR and ESI-MS spectra. Compounds **a**, **b** and **c** were prepared according to our previous work [12,38,39,40].

#### 3.2.1. Preparation of Rhodamine-B hydrazide (**1**)

Rhodamine B (2.0 g) was dissolved in ethanol (50 mL). Hydrazine hydrate (2.5 mL) was then added dropwise with vigorous stirring at room temperature. After the addition, the stirred solution was allowed to reflux for about 6–8 h. The solution changed from dark pink to light orange. Then the mixture was cooled and solvent was removed under reduced pressure. HCl (1 M, about 50 mL) was added to the mixture in the flask to generate a clear red solution. After that, 1 M NaOH was added slowly with stirring until the pH of the solution reached 9–10. The resulting precipitate was filtered and washed 4–5 times with water (15 mL each time). After drying under reduced pressure, the reaction yielded 1.8 g (95%) of **1** as a pink solid. Mp: 176–178 °C; FT-IR (KBr), *ν*, cm^−1^: 1614 (C=O), 1379, 1118 (C−N), 1224, 1015 (C−O); ^1^H-NMR (CDCl_3_), *δ* (ppm): 1.16 (12H, t, *J* = 6.5 Hz), 3.34 (8H, d, *J* = 6.5 Hz), 3.62 (2H, s), 6.29 (2H, d, *J* = 7.5 Hz), 6.42–6.47 (4H, m), 7.11 (1H, s), 7.45 (2H, s), 7.93 (1H, s); ^13^C-NMR (CDCl_3_), *δ* (ppm): 12.6, 44.4, 65.9, 98.0, 104.6, 108.1, 123.0, 123.8, 128.1, 130.0, 132.5, 148.9, 151.5, 153.8, 166.1. ESI-MS *m/z*: Calcd for C_28_H_32_N_4_O_2_ [M + H]^+^: 457.2604, found: 457.2500.

#### 3.2.2. Preparation of Compounds **L1**–**L3**

Rhodamine hydrazide (**1**, 0.23 g, 0.5 mmol) and dialdehyde **a**, **b** or **c** (0.25 mmol) were dissolved in absolute ethanol (20 mL) and the reaction mixture was refluxed for 24 h. The obtained solid was filtered and washed three times with ethanol (10 mL). The product was dried under vacuum, affording pink solids of **L1**–**L3**, respectively.

*IUPAC name* (**L1**). Yield: 0.14 g (48%); Mp: 187–189 °C; FT-IR (KBr), *ν*, cm^−1^: 1616 (C=O), 1309, 1116 (C−N), 1226 (C−O); ^1^H-NMR (CDCl_3_), *δ* (ppm): 1.04 (24H, t, *J* = 6.5 Hz), 3.20 (16H, s), 4.18 (4H, s), 6.22 (4H, d, *J* = 8.5 Hz), 6.39 (4H, s), 6.54 (4H, d, *J* = 8.5 Hz), 6.95 (2H, t, *J* = 7.5 Hz), 7.00 (2H, d, *J* = 8.5 Hz), 7.07 (2H, d, *J* = 6.5 Hz), 7.31 (2H, t, *J* = 7.5 Hz), 7.46 (4H, qn, *J* = 7.0 Hz), 8.01 (4H, d, *J* = 7.0 Hz), 8.69 (2H, s); ^13^C-NMR (CDCl_3_), *δ* (ppm): 12.5, 44.2, 65.5, 66.2, 97.8, 105.6, 108.1, 112.3, 121.2, 123.4, 123.7, 124.2, 126.4, 128.1, 128.2, 128.6, 130.8, 133.3, 141.4, 148.9, 152.5, 152.8, 157.0, 165.1. ESI-MS *m/z*: Calcd for C_72_H_74_N_8_O_6_ [M + Na]^+^: 1169.5629, found: 1169.5682.

*IUPAC name* (**L2**). Yield: 0.17 g (58%); Mp: 156–158 °C; FT-IR (KBr), *ν*, cm^−1^: 1614 (C=O), 1306, 1112 (C−N), 1227 (C−O); ^1^H-NMR (CDCl_3_), *δ* (ppm): 1.09 (24H, t, *J* = 6.5 Hz), 2.05 (2H, s), 3.25 (16H, d, *J* = 7.0 Hz), 3.99 (4H, s), 6.23 (4H, d, *J* = 8.0 Hz), 6.41 (4H, s), 6.56 (4H, d, *J* = 8.0 Hz), 6.86 (4H, d, *J* = 8.0 Hz), 7.07 (2H, d, *J* = 7.0 Hz), 7.21 (2H, t, *J* = 7.0 Hz), 7.44 (4H, t, *J* = 7.0 Hz), 7.93 (2H, d, *J* = 7.5 Hz), 7.99 (2H, d, *J* = 6.5 Hz), 8.80 (2H, s); ^13^C-NMR (CDCl_3_), *δ* (ppm): 12.6, 29.5, 44.2, 64.8, 65.6, 98.0, 106.0, 108.1, 112.0, 120.6, 123.4, 123.6, 124.0, 126.5, 127.8, 128.1, 128.6, 130.7, 133.2, 142.6, 148.9, 152.4, 152.8, 157.5, 165.2. ESI-MS *m/z*: Calcd for C_73_H_76_N_8_O_6_Na^+^ [M + Na]^+^: 1183.5786, found: 1183.5770.

*IUPAC name* (**L3**). Yield: 0.18 g (60%); Mp: 224–226 °C; FT-IR (KBr), *ν*, cm^−1^: 1614 (C=O), 1303, 1115 (C−N), 1227 (C−O); ^1^H-NMR (CDCl_3_), *δ* (ppm): 1.12 (24H, t, *J* = 6.5 Hz), 3.28 (16H, d, *J* = 7.0 Hz), 3.83 (4H, s), 4.01 (4H, s), 6.22 (4H, d, *J* = 8.5 Hz), 6.43 (4H, s), 6.54 (4H, d, *J* = 9.0 Hz), 6.77 (2H, d, *J* = 8.0 Hz), 6.85 (2H, d, *J* = 7.5 Hz), 7.10–7.16 (4H, m), 7.46 (4H, qn, *J* = 7.5 Hz), 7.84 (2H, d, *J* = 7.5 Hz), 7.97 (2H, d, *J* = 7.0 Hz), 9.06 (2H, s); ^13^C-NMR (CDCl_3_), *δ* (ppm): 12.6, 44.3, 65.9, 68.3, 69.7, 98.0, 106.3, 108.0, 113.0, 121.0, 123.3, 123.8, 124.5, 126.3, 127.9, 128.1, 129.4, 130.8, 133.1, 143.8, 148.8, 151.9, 153.2, 157.4, 164.8. ESI-MS *m/z*: Calcd for C_74_H_78_N_8_O_7_ [M + Na]^+^: 1213.5891, found: 1213.5852.

## 4. Conclusions

The newly synthesized fluoroionophores **L1**–**L3** based on rhodamine exhibit good selectivity and sensitivity toward Al^3+^ ion over other tested metal ions in MeOH−DMSO (99:1 v/v). They exhibited a reversible absorption and fluorescence enhancement toward Al(III) via a 1:1 binding mode at neutral pH. A polymeric thin film can be prepared by doping PVC with a probe **L1**–**L3**. Such a thin layer can be used as a sensor to detect Al^3+^ with high selectivity.

## Supplementary Files

Supplementary File 1

## References

[1] Baylor N.W., Egan W., Richman P. (2002). Aluminum salts in vaccines-US perspective. Vaccine.

[2] Soni M.G., White S.M., Flamm W.G., Burdock G.A. (2001). Safety evaluation of dietary aluminum. Regul. Toxicol. Pharm..

[3] Banks W.A., Kastin A.J. (1989). Aluminum-induced neurotoxicity: Alterations in membrane function at the blood-brain barrier. Neurosci. Biobehav. Rev..

[4] Good P.F., Olanow C.W., Perl D.P. (1992). Neuromelanin-containing neurons of the substantia nigra accumulate iron and aluminum in parkinson’s disease: A LAMMA study. Brain Res..

[5] Kawahara M., Muramoto K., Kobayashi K., Mori H., Kuroda Y. (1994). Aluminum promotes the aggregation of alzheimer’s amyloid β-protein *in vitro*. Biochem. Biophys. Res. Commun..

[6] Paik S.R., Lee J.H., Kim D.H., Chang C.S., Kim J. (1997). Aluminum-induced structural alterations of the precursor of the non-Aβ component of alzheimer’s disease amyloid. Arch. Biochem. Biophys..

[7] Lin J.L., Kou M.T., Leu M.L. (1996). Effect of long-term low-dose aluminum-containing agents on hemoglobin synthesis in patients with chronic renal insufficiency. Nephron.

[8] Alvim M.N., Ramos F.T., Oliveira D.C., Isaias R.M.S., Franca M.G.C. (2012). Aluminium localization and toxicity symptoms related to root growth inhibition in rice (Oryza sativa L.) seedlings. J. Biosci..

[9] Alamgir A.N.M., Akhter S. (2009). Effects of aluminium (Al^3+^) on seed germination and seedling growth of wheat (Triticum aestivum L.). Bangladesh J. Bot..

[10] Gupta V.K., Jain A.K., Maheshwari G. (2007). Aluminum(III) selective potentiometric sensor based on morin in poly(vinyl chloride) matrix. Talanta.

[11] Gupta V.K., Goyal R.N., Jain A.K., Sharma R.A. (2009). Aluminium (III)-selective PVC membrane sensor based on a schiff base complex of N,N'-bis (salicylidene)-1,2-cyclohexanediamine. Electrochim. Acta.

[12] Gupta V.K., Singh A.K., Ganjali M.R., Norouzi P., Faridbod F., Mergu N. (2013). Comparative study of colorimetric sensors based on newly synthesized schiff bases. Sens. Actuators B Chem..

[13] Chen S., Fang Y.M., Xiao Q., Li J., Li S.B., Chen H.J., Sun J.J., Yang H.H. (2012). Rapid visual detection of aluminium ion using citrate capped gold nanoparticle. Analyst.

[14] Mohadesi A., Taher M.A. (2007). Voltammetric determination of Cu(II) in natural waters and human hair at a meso-2,3-dimercaptosuccinic acid self-assembled gold electrode. Talanta.

[15] Mashhadizadeh M.H., Pesteh M., Talakesh M., Sheikhshoaie I., Ardakani M.M., Karimi M.A. (2008). Solid phase extraction of lead(II), copper(II), cadmium(II) and nickel(II) using gallic acid-modified silica gel prior to determination by flame atomic absorption spectrometry. Spectrochim. Acta B.

[16] Cassella R.J., Magalhaes O.I.B., Couto M.T., Lima E.L.S., Neves M.A.F.S., Coutinho F.M.B. (2005). Synthesis and application of a functionalized resin for flow injection/ F AAS copper determination in waters. Talanta.

[17] Ali A., Shen H., Yin X. (1998). Simultaneous determination of trace amounts of nickel, copper and mercury by liquid chromatography coupled with flow injection online derivatization and preconcentration. Anal. Chim. Acta.

[18] Ferreira S.L.C., Queiroz A.S., Fernandes M.S., dos Santos H.C. (2002). Application of factorial designs and doehlert matrix in optimization of experimental variables associated with the preconcentration and determination of vanadium and copper in seawater by inductively coupled plasma optical emission spectrometry. Spectrochim. Acta B.

[19] Li Y.P., Liu X.M., Zhang Y.H., Chang Z. (2013). A fluorescent and colorimetric sensor for Al^3+^ based on a dibenzo-18-crown-6 derivative. Inorg. Chem. Commun..

[20] Chen C.H., Liao D.J., Wan C.F., Wu A.T. (2013). A turn-on and reversible schiff base fluorescence sensor for Al^3+^ ion. Analyst.

[21] Gupta V.K., Singh A.K., Mergu N. (2014). Antipyrine based schiff bases as turn-on fluorescent sensors for Al(III) ion. Electrochim. Acta.

[22] Gupta V.K., Singh A.K., Kumawat L.K. (2014). Thiazole schiff base turn-on fluorescent chemosensor for Al^3+^ ion. Sens. Actuators B Chem..

[23] Gupta V.K., Mergu N., Singh A.K. (2014). Fluorescent chemosensors for Zn^2+^ ions based on flavonol derivatives. Sens. Actuators B Chem..

[24] Gupta V.K., Mergu N., Kumawat L.K., Singh A.K. (2015). Selective naked-eye detection of magnesium (II) ions using a coumarin-derived fluorescent probe. Sens. Actuators B Chem..

[25] Kim K.B., You D.M., Jeon J.H., Yeon Y.H., Kim J.H., Kim C. (2014). A fluorescent and colorimetric chemosensor for selective detection of aluminum in aqueous solution. Tetrahedron Lett..

[26] Azadbakht R., Khanabadi J. (2013). A novel aluminum-sensitive fluorescent nano-chemosensor based on naphthalene macrocyclic derivative. Tetrahedron.

[27] Zhou D., Sun C., Chen C., Cui X., Li W. (2015). Research of a highly selective fluorescent chemosensor for aluminum(III) ions based on photoinduced electron transfer. J. Mol. Struct..

[28] Soroka K., Vithanage R.S., Phillips D.A., Walker B., Dasgupta P.K. (1987). Fluorescence properties of metal complexes of 8-hydroxyquinoline-5-sulfonic acid and chromatographic applications. Anal. Chem..

[29] Kim H.N., Lee M.H., Kim H.J., Kim J.S., Yoon J. (2008). A new trend in rhodamine-based chemosensors: application of spirolactam ring-opening to sensing ions. Chem. Soc. Rev..

[30] Beija M., Afonso C.A.M., Martinho J.M.G. (2009). Synthesis and applications of Rhodamine derivatives as fluorescent probes. Chem. Soc. Rev..

[31] Grynkiewicz G., Poenie M., Tsien R.Y. (1985). A new generation of Ca^2+^ indicators with greatly improved fluorescence properties. J. Biol. Chem..

[32] Minta A., Tsien R.Y. (1989). Fluorescent indicators for cytosolic sodium. J. Biol. Chem..

[33] Yu C., Zhang J., Wang R., Chen L. (2010). Highly sensitive and selective colorimetric and off-on fluorescent probe for Cu^2+^ based on rhodamine derivative. Org. Biomol. Chem..

[34] Kaewtong C., Wanno B., Uppa Y., Morakot N., Pulpoka B., Tuntulani T. (2011). Facile synthesis of rhodamine-based highly sensitive and fast responsive colorimetric and off-on fluorescent reversible chemosensors for Hg^2+^: Preparation of fluorescent thin film sensor. Dalton Trans..

[35] Lee M.H., Wu J.S., Lee J.W., Jung J.H., Kim J.S. (2007). Highly sensitive and selective chemosensor for Hg^2^^+^ based on the rhodamine fluorophore. Org. Lett..

[36] Bag B., Pal A. (2011). Rhodamine-based probes for metal ion-induced chromo-/fluorogenic dual signalling and their selectivity towards Hg(II) ion. Org. Biomol. Chem..

[37] Shafiee A., Salleh M.M., Yahaya M. (2011). Determination of HOMO and LUMO of [6,6]-phenyl C61-butyric acid 3-ethylthiophene ester and poly (3-octyl-thiophene-2,5-diyl) through voltametry characterization. Sains Malays..

[38] Gupta V.K., Singh A.K.,  Mergu N. (2012). A new beryllium ion-selective membrane electrode based on dibenzo(perhydrotriazino)aza-14-crown-4 ether. Anal. Chim. Acta.

[39] Xiang Y., Tong A., Jin P., Ju Y. (2006). New fluorescent rhodamine hydrazone chemosensor for Cu(II) with high selectivity and sensitivity. Org. Lett..

[40] Gupta V.K., Ganjali M.R., Norouzi P., Khani H., Nayak A., Agarwal S (2011). Electrochemical Analysis of some Toxic Metals and Drugs by Ion Selective Electrodes. Crit. Rev. Anal. Chem..

