# Supplementary material for: Highly Sensitive and Selective Colorimetric and Off-On Fluorescent Reversible Chemosensors for Al3+ Based on the Rhodamine Fluorophore
Source: Sensors (Basel). 2015 Apr 17;15(4):9097–111. doi: 10.3390/s150409097 (PMC4431275; doi:10.3390/s150409097)
Supplement: Supplementary File 1 [file sensors-15-09097-s001.pdf]

OPEN ACCESS

sensors

ISSN 1424-8220

www.mdpi.com/journal/sensors

Supplementary Information

Highly Sensitive and Selective Colorimetric and Off-On Fluorescent Reversible Chemosensors for Al^{3+} Based on the Rhodamine Fluorophore. *Sensors* 2015, *15*, 9097-9111

Naveen Mergu [1], Ashok Kumar Singh [1] and Vinod Kumar Gupta [1,2,3,*]

1 Department of Chemistry, Indian Institute of Technology Roorkee, Roorkee 247 667, India; E-Mails: mergu.naveen@gmail.com (N.M.); akscyfcy@gmail.com (A.K.S.)

2 Center for Environment and Water, The research Institute, King Fahd University of Petroleum & Minerals, Dhahran 31261, Saudi Arabia

3 Department of Applied Chemistry, University of Johansburg, Johannesburg 17011, South Africa

* Author to whom correspondence should be addressed; E-Mails: vinodfcy@iitr.ac.in or vinodfcy@gmail.com; Tel.: +91-133-2285-801; Fax: +91-133-2273-560.

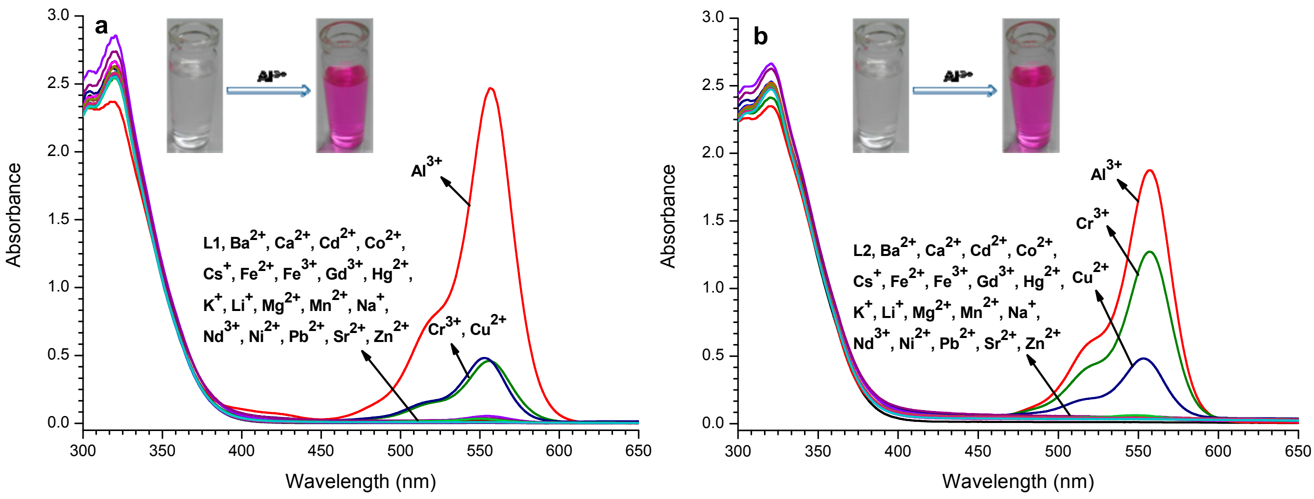

Figure S1. Absorbance spectra of **L1** (**a**, 50 µM) and **L2** (**b**, 50 µM) in presence of various metal ions (50 µM) in MeOH−DMSO (99:1 v/v). Inset: Visual color change of probe upon addition of Al^{3+}.

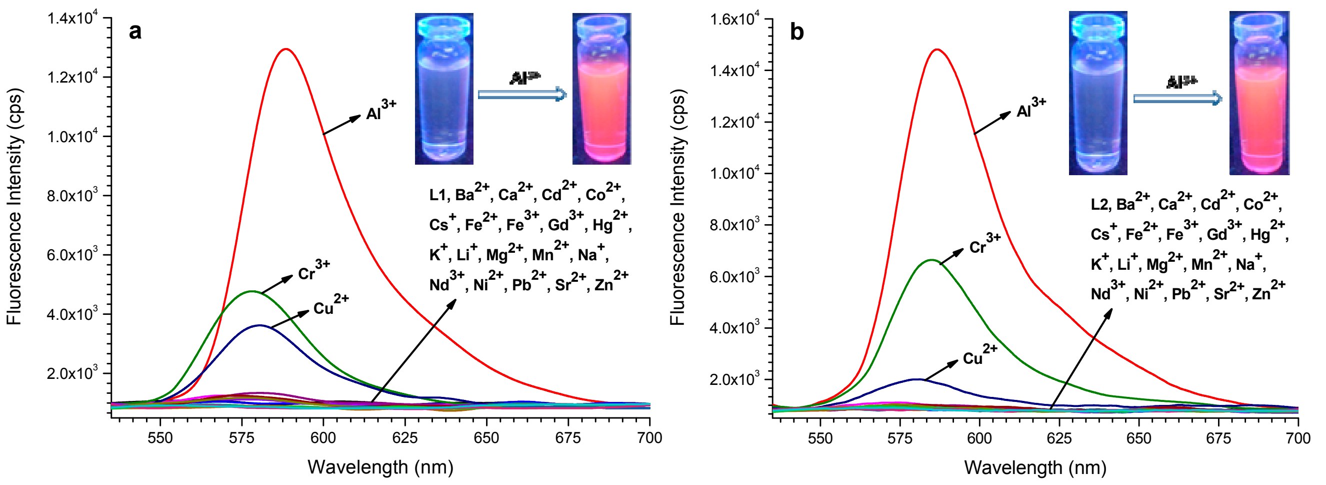

Figure S2. Fluorescence spectra (λ_{ex} = 520 nm) of **L1** (**a**, 50 μM) and **L2** (**b**, 50 μM) in presence of various metal ions (50 μM) in MeOH−DMSO (99:1 v/v). Inset: Visual color change of probe upon addition of Al^{3+}.

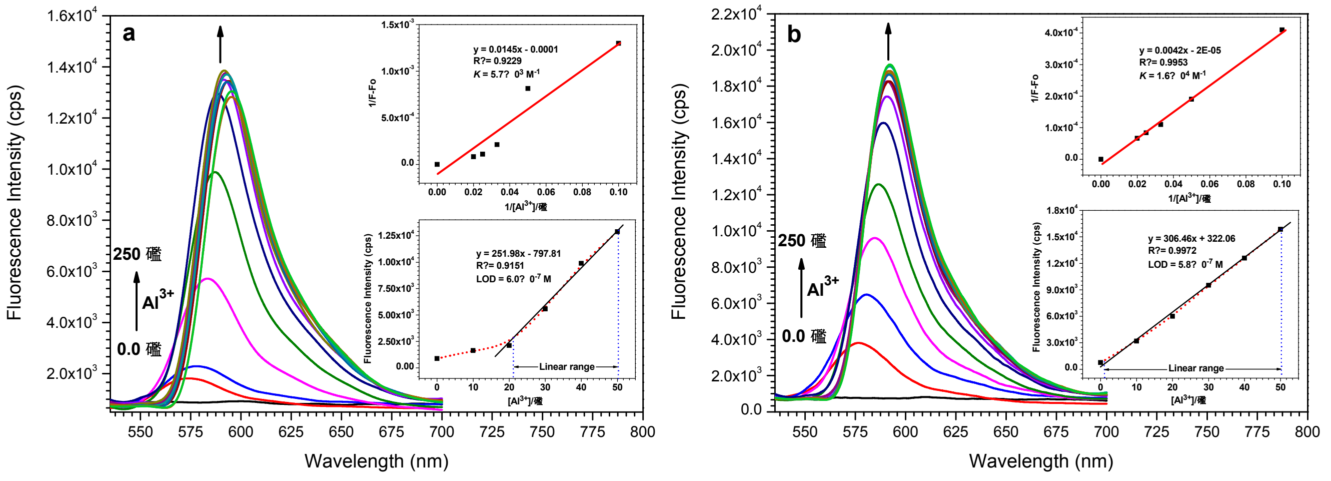

Figure S3. The fluorescence emission spectral pattern of **L1** (**a**) and **L2** (**b**) in the presence of increasing concentrations of Al^{3+} (0, 10, 20, 30, 40, 50, 75, 100, 125, 150, 175, 200, 225, 250 μM). Inset: Linear regression plot of fluorescence intensity change $1/(F\text{-}F_0)$ as a function of concentration $1/[Al^{3+}]$ (top), fluorescence enhancement change as a function of concentration of Al(III) added (bottom).

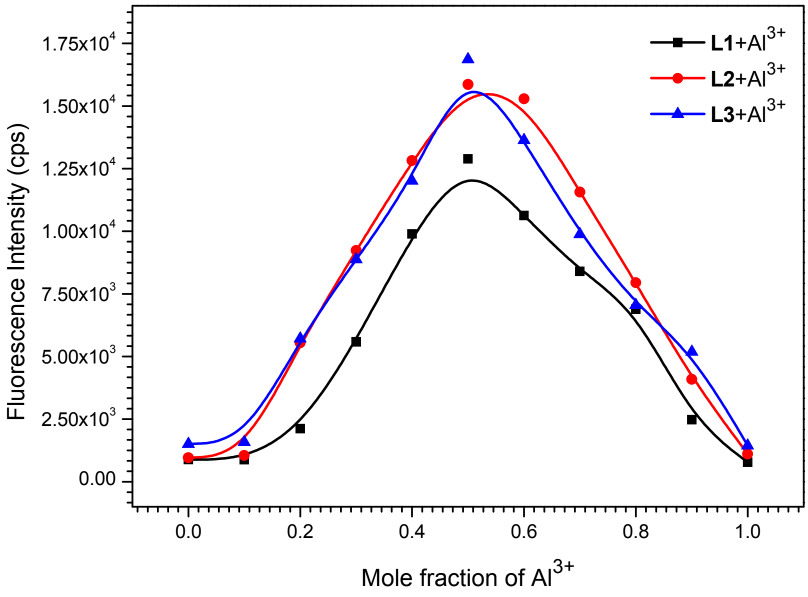

Figure S4. Job's plot for **L1–L3** with Al^{3+}, fluorescence intensity at 587 nm was plotted as a function of the molar ratio.

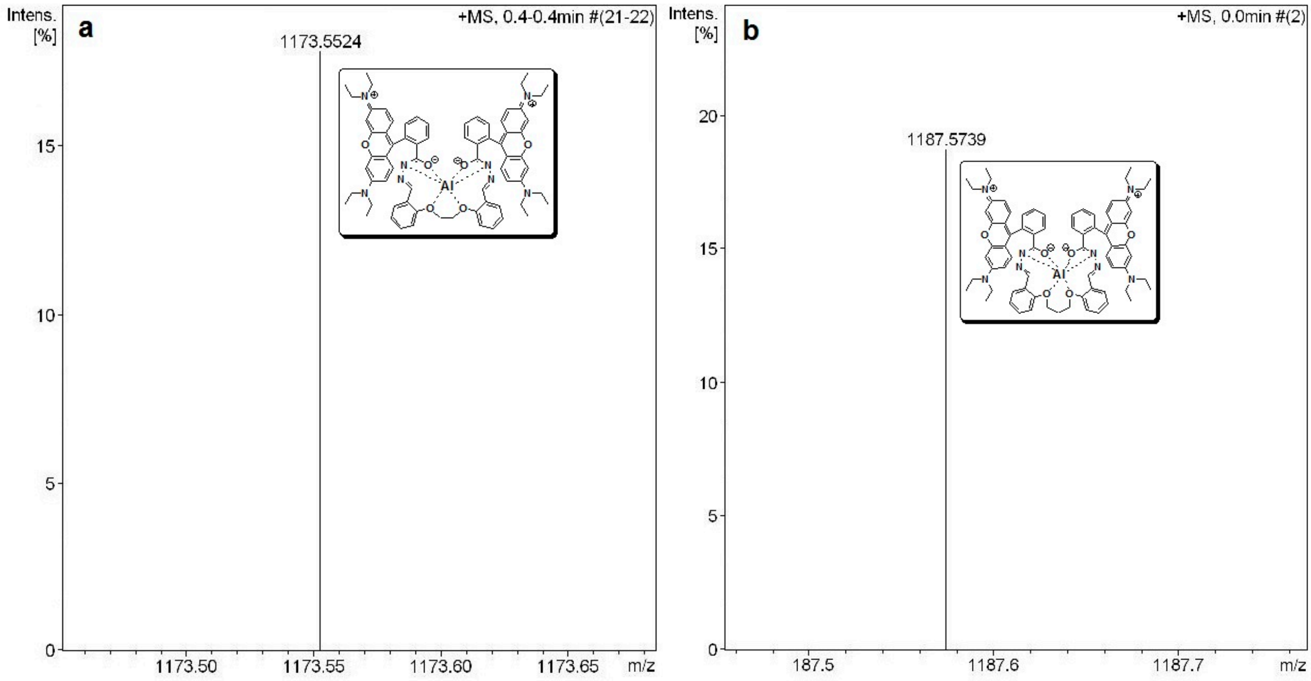

Figure S5. ESI-MS spectrum of **L1** (**a**) and **L2** (**b**) upon addition of $AlCl_3.6H_2O$ (1.0 eqiuv.) in MeOH.

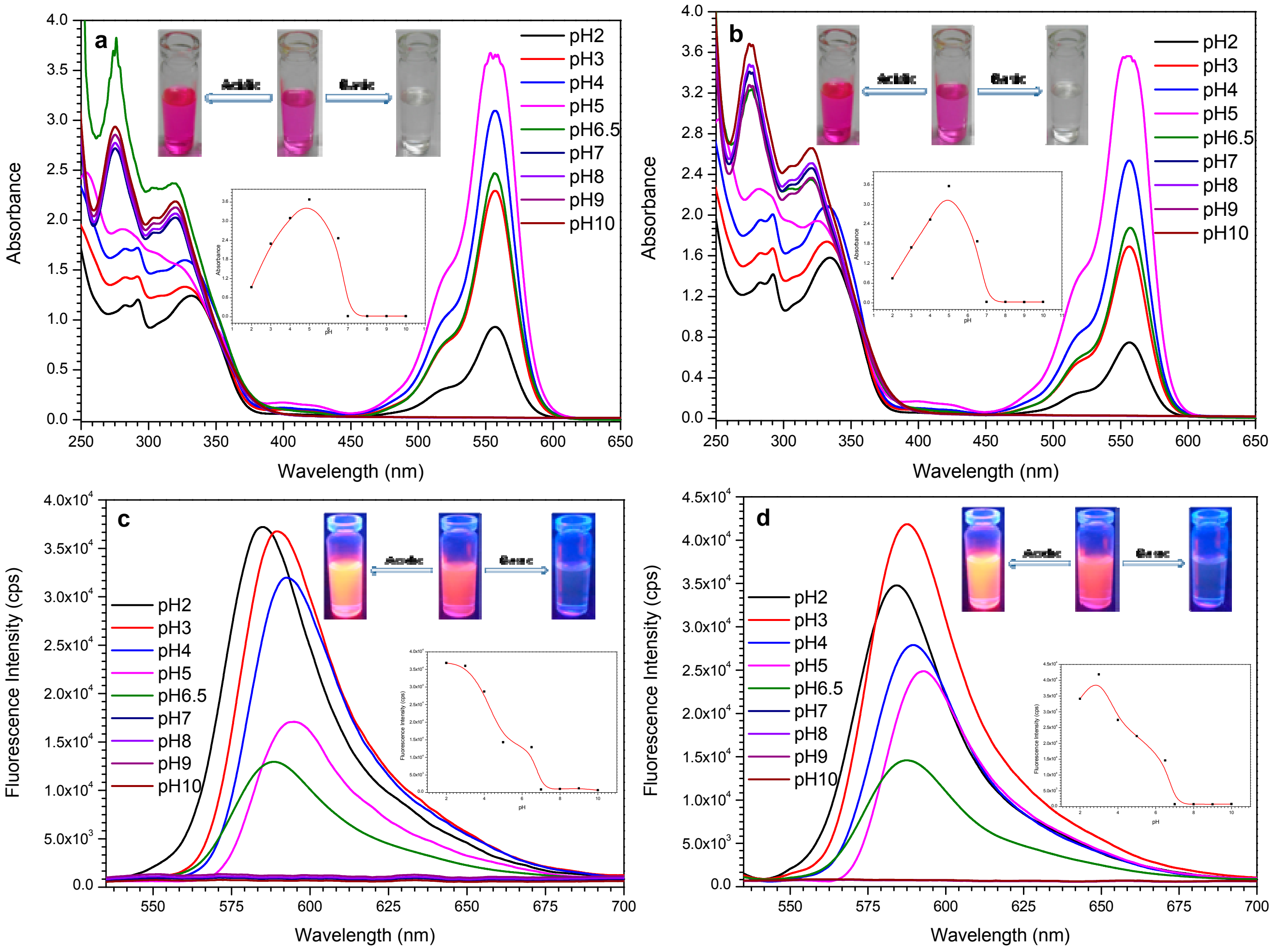

Figure S6. UV-vis absorbance (**a**,**b**) and Fluorescence emission (**c**,**d**) spectral changes of **L1** and **L2** with Al^{3+} as a function of pH. Inset: Color changes of probe + Al^{3+} in different pH media under a normal (a and b) and UV (c and d) lamp (top), absorbance (a and b, at 557 nm) and emission (c and d, at 587 nm) intensities of **L1** and **L2** in the presence of Al^{3+} with pH variation (bottom).

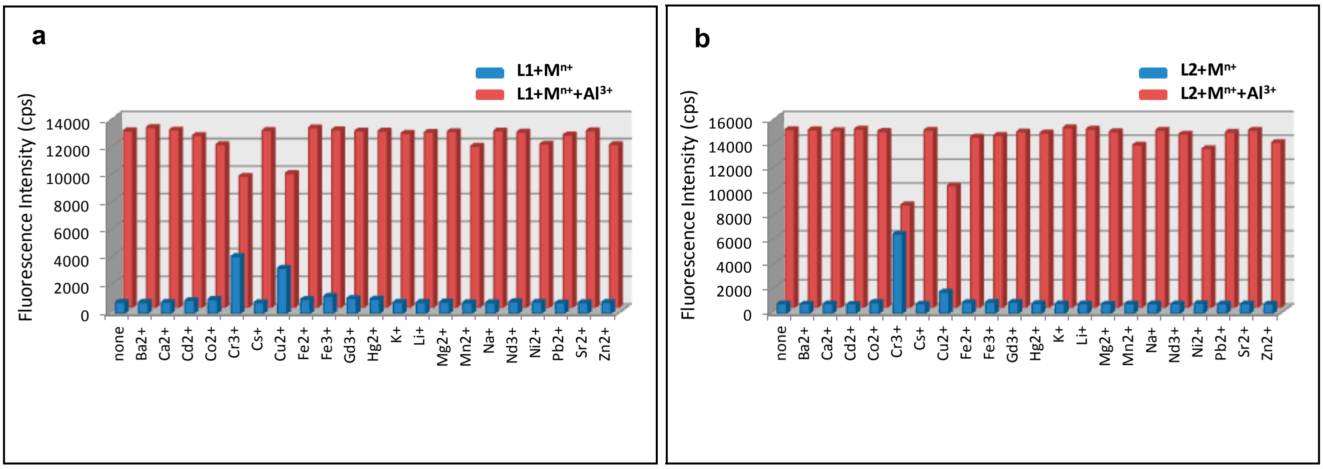

Figure S7. Competitive selectivity of probes **L1** (**a**) and **L2** (**b**) toward various metal ions (1.0 equiv.) in the absence (blue bars) and presence (red bars) of Al^{3+} (1.0 equiv.) with an excitation of 520 nm.

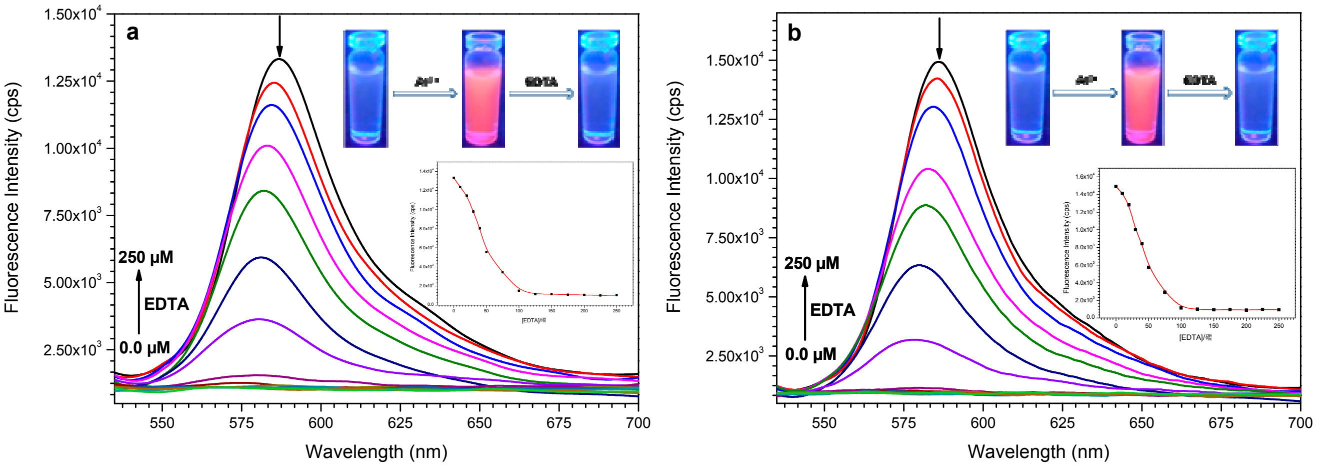

Figure S8. The variation in fluorescence emission spectra of **L1** + Al^{3+} (**a**) and **L2** + Al^{3+} (**b**) upon addition of EDTA (0, 10, 20, 30, 40, 50, 75, 100, 125, 150, 175, 200, 225, 250 μM). Inset: Color changes of probe+Al^{3+} upon addition of EDTA (1.0 equiv.) (top), fluorescence spectral changes at 587 nm as a function of the amount of EDTA (bottom).

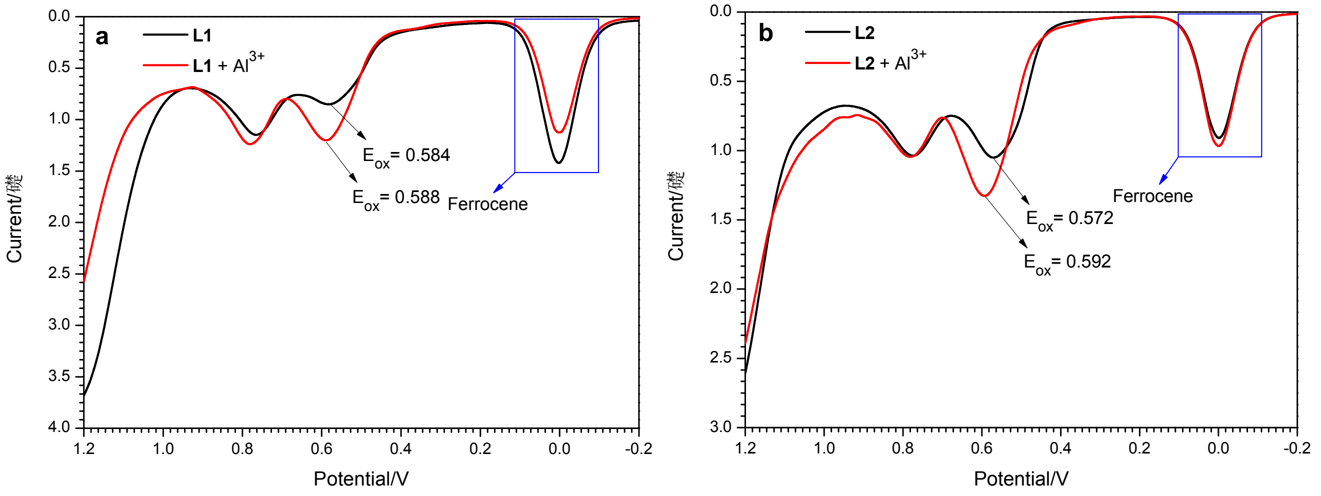

Figure S9. Differential pulse voltammograms recorded for **L1** and **L2**, and the corresponding Al^{3+} addition products in MeOH−DMSO (99:1 v/v).

^{1}H NMR Titration

Both doublet and triplet of H_j and H_l, respectively, were shifted upfield then they were combined with each other and gave a simple multiplet at about 6.8 ppm, while a combine signal of H_b, H_d and H_i was splitted into two signals of H_i and a combine signal of H_b and H_d. The distance between two signals of H_c and H_k of fluoroionophore **L3** is also varied upon addition of Al^{3+}. Other aryl-proton (H_a) of rhodamine moiety was also shifted slightly downfield because the strong coordination between **L3** and Al^{3+} ion.

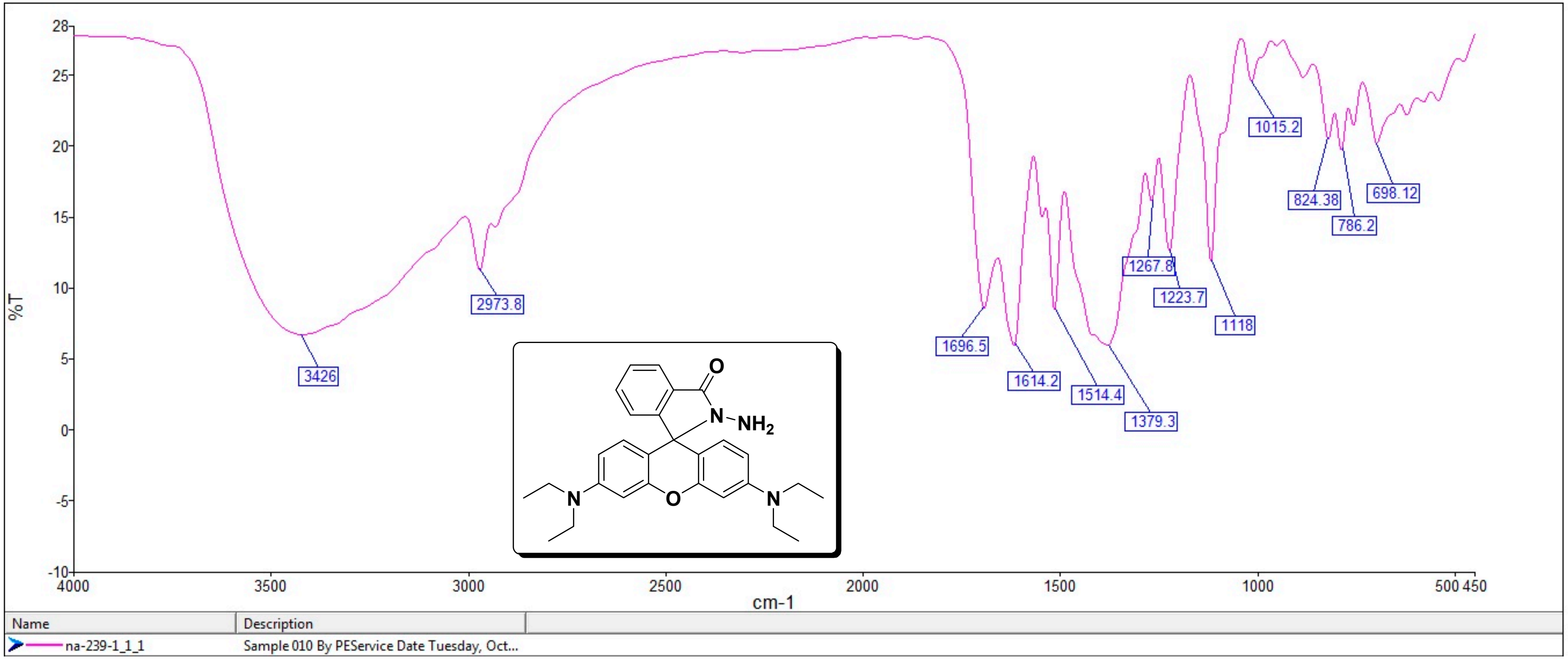

Figure S10. FT-IR Spectrum (KBr) of **1**.

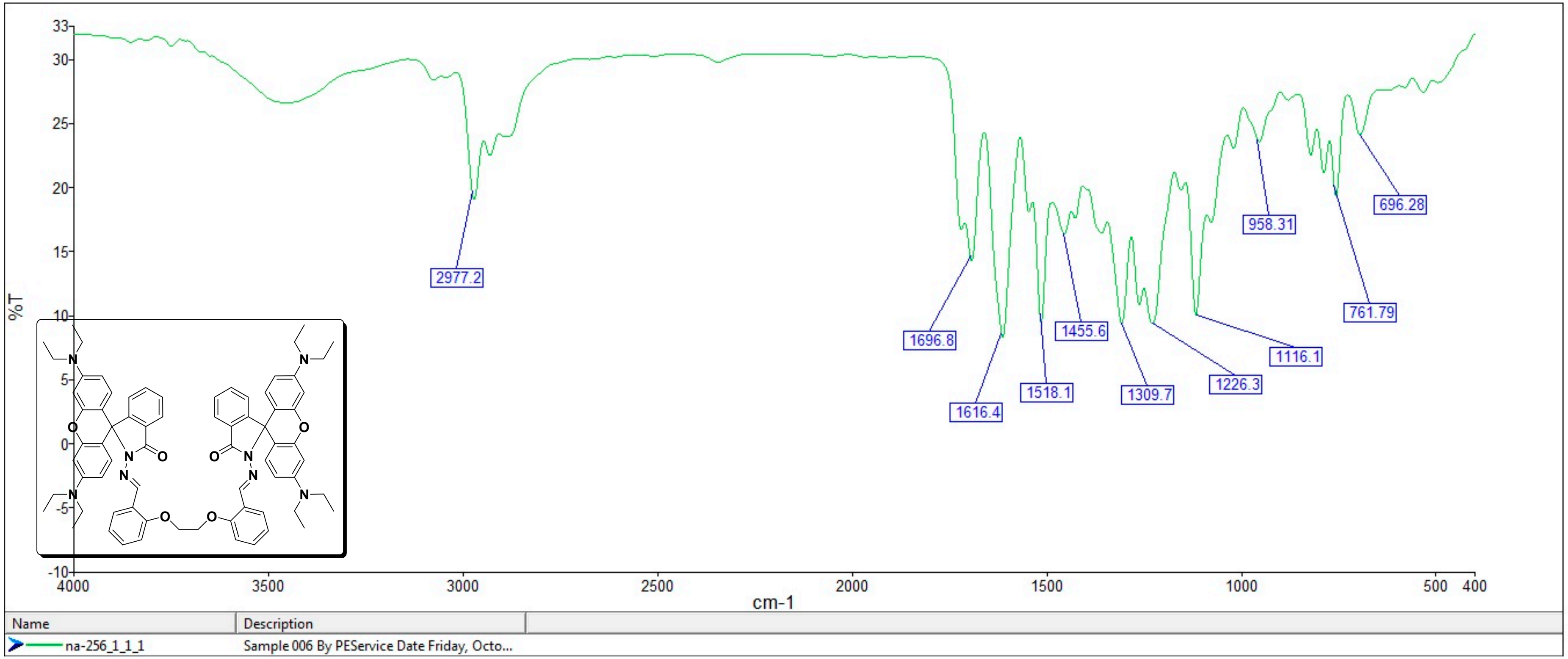

Figure S11. FT-IR Spectrum (KBr) of **L1**.

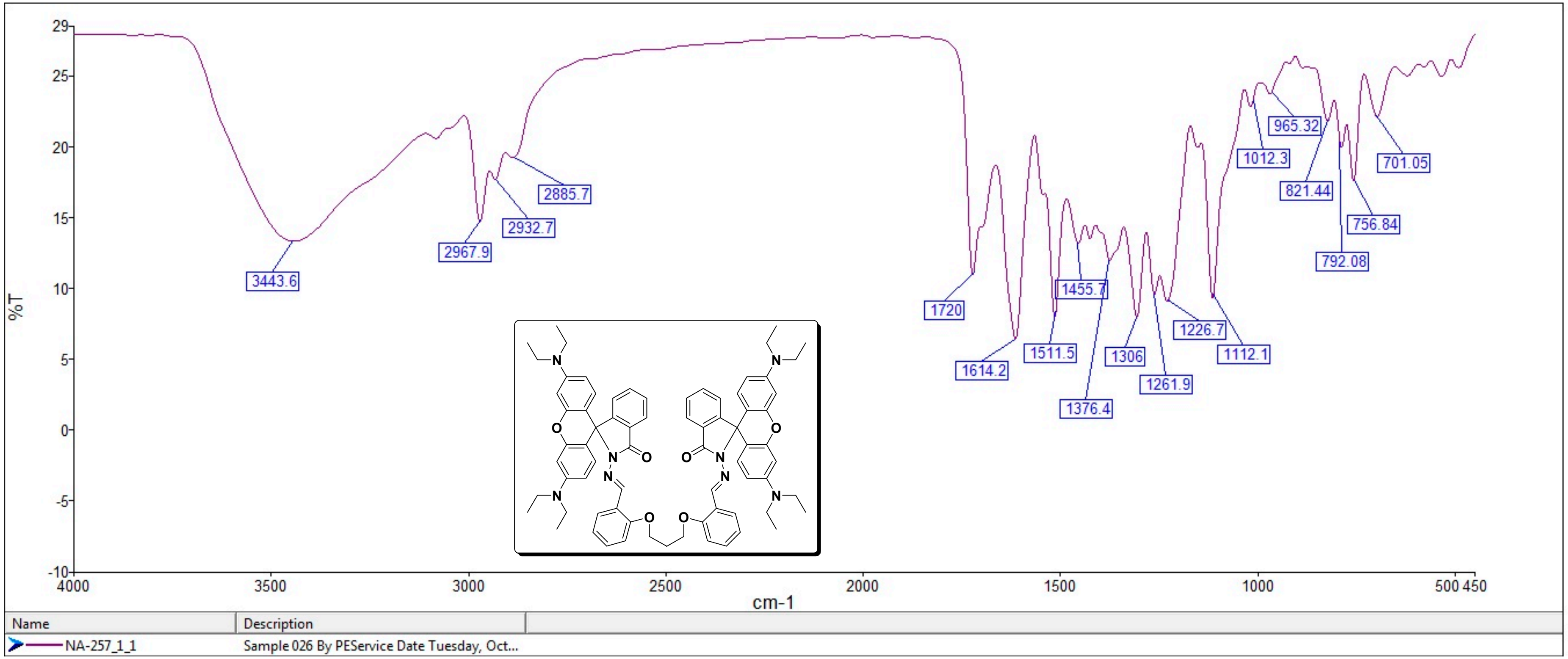

Figure S12. FT-IR Spectrum (KBr) of **L2**.

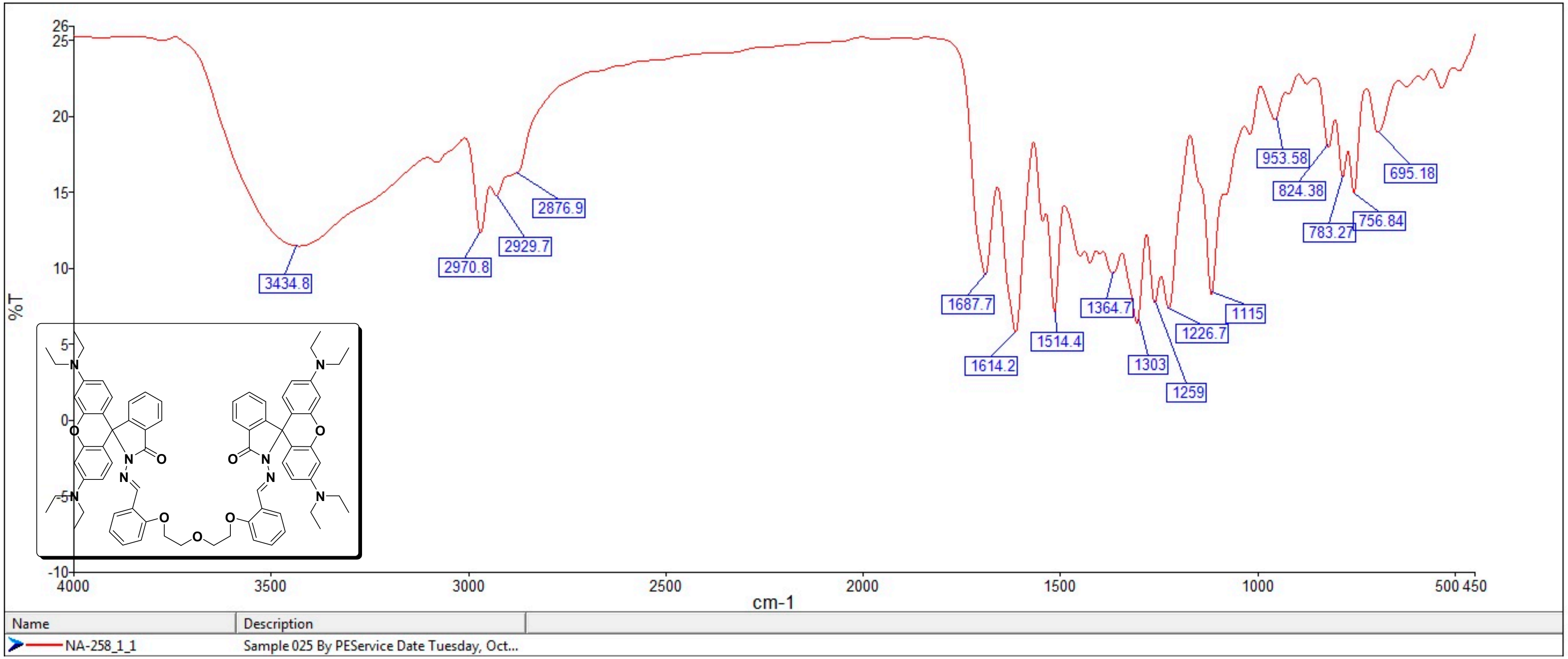

Figure S13. FT-IR Spectrum (KBr) of **L3**.

Detection Limit

The detection limit was carried out by the following calculations:

$$LOD = 3\sigma/m$$

where, "σ" is the standard deviation of probe (without metal) and "m" is the slope of the plot of fluorescence emission vs concentration of metal.

From the Figure 2b: σ is calculated to be 46.05 and m is 273.44.

$LOD = (3 \times 46.05)/273.44$

$LOD = 0.5\ \mu M$.

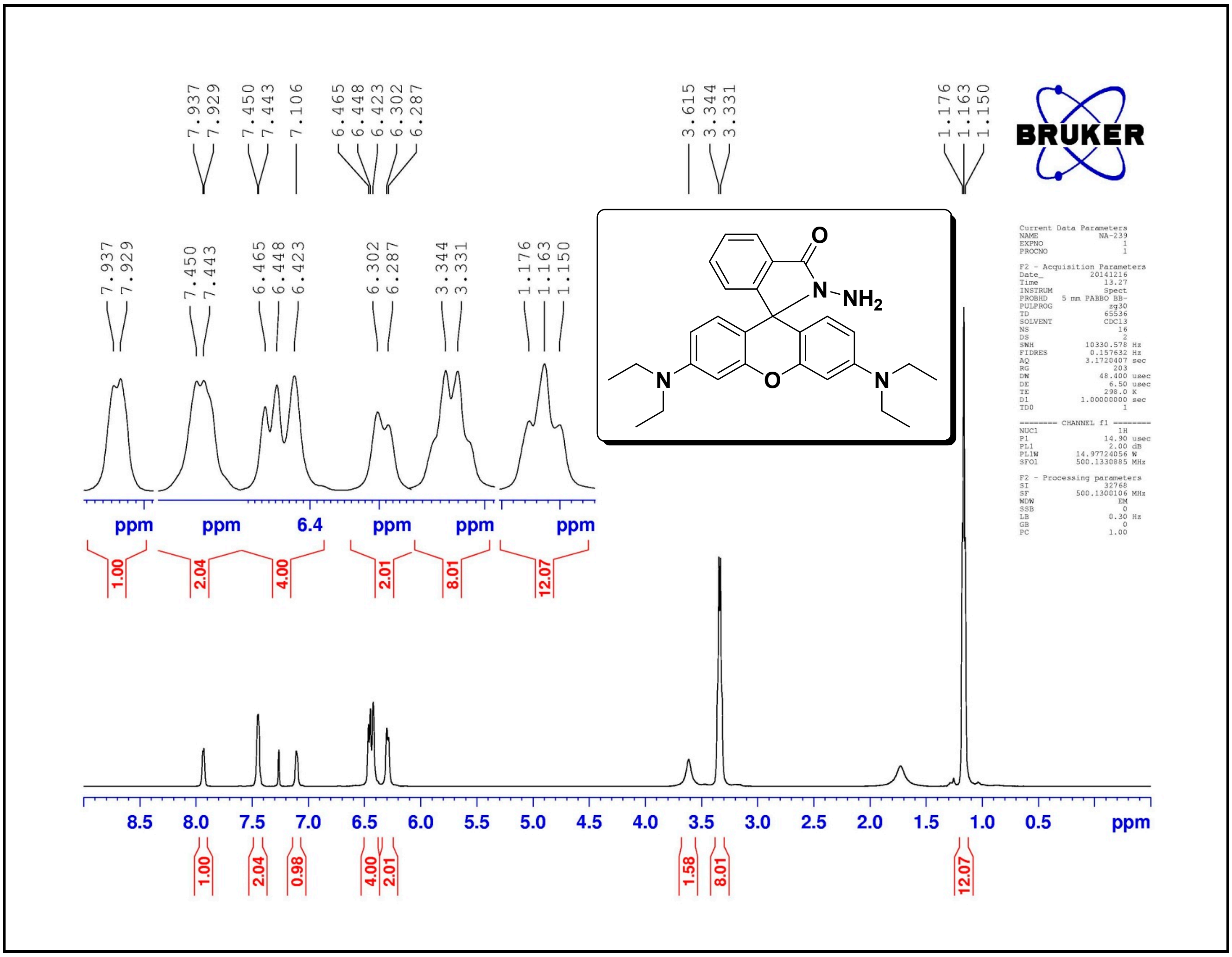

Figure S14. ^{1}H NMR Spectrum (500 MHz, $CDCl_3$) of **1**.

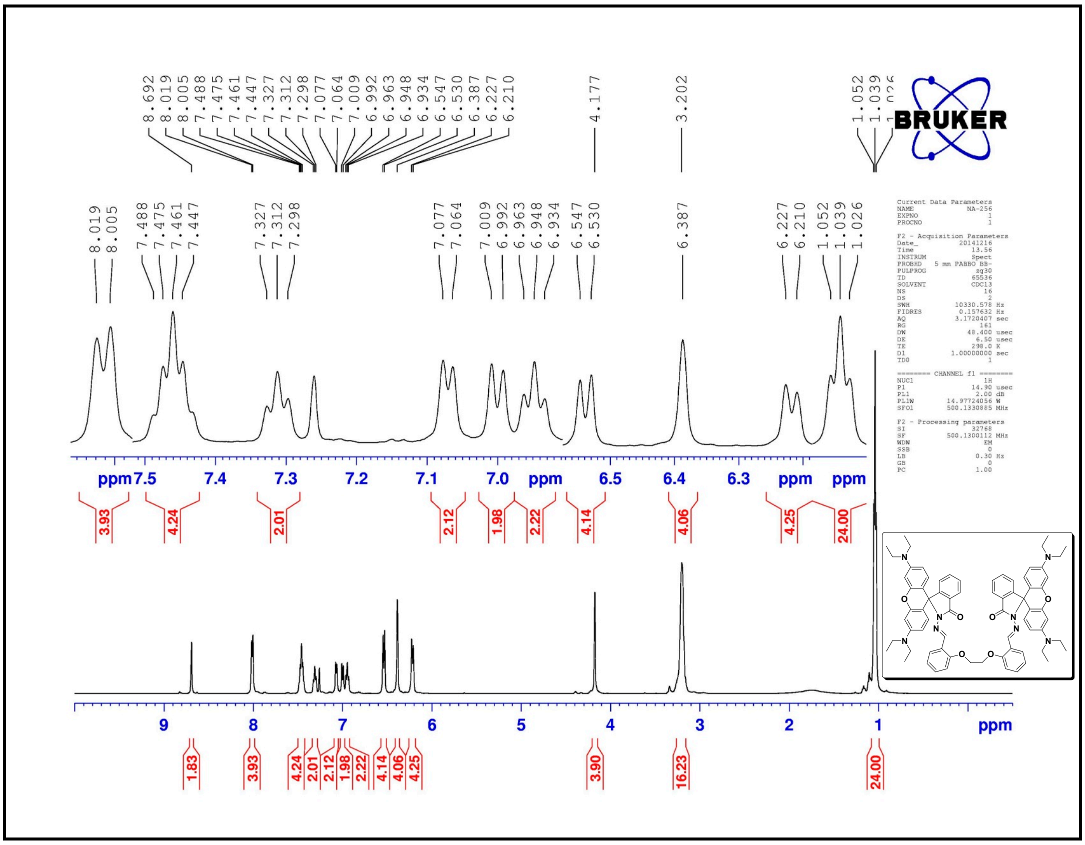

Figure S15. ^{1}H NMR Spectrum (500 MHz, $CDCl_3$) of **L1**.

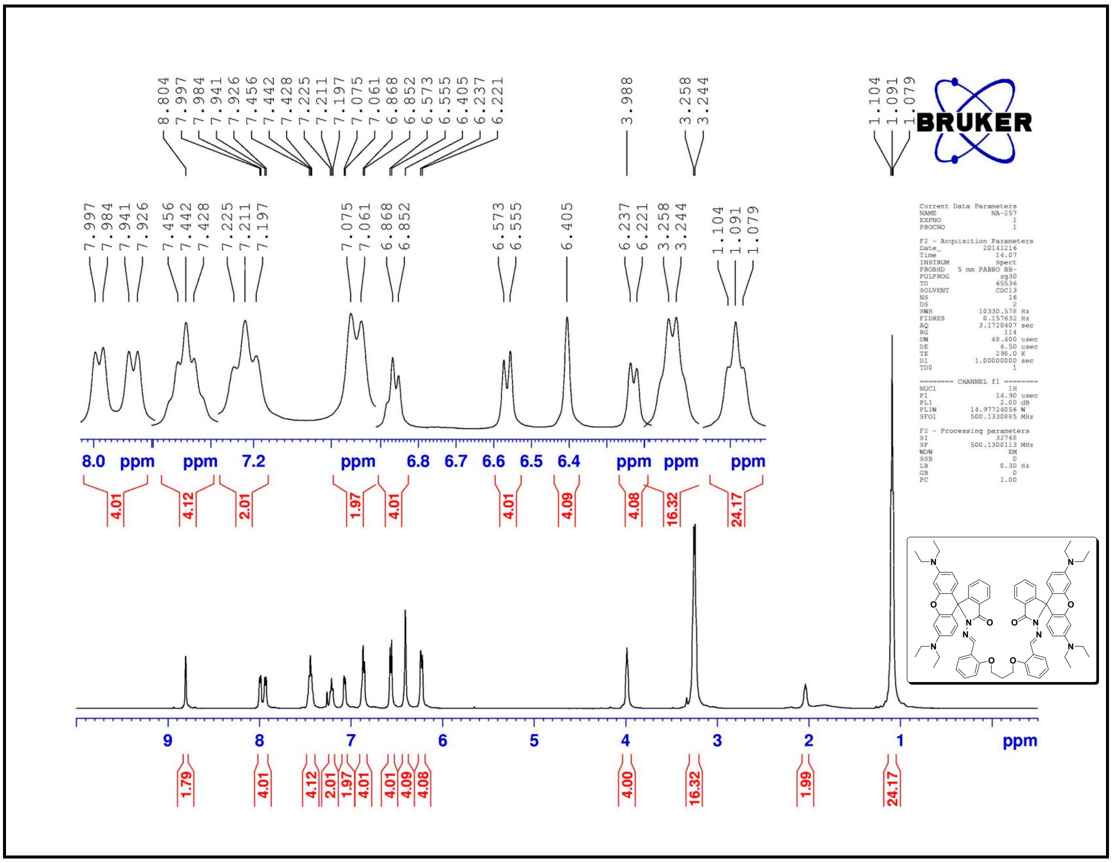

Figure S16. ^{1}H NMR Spectrum (500 MHz, $CDCl_3$) of **L2**.

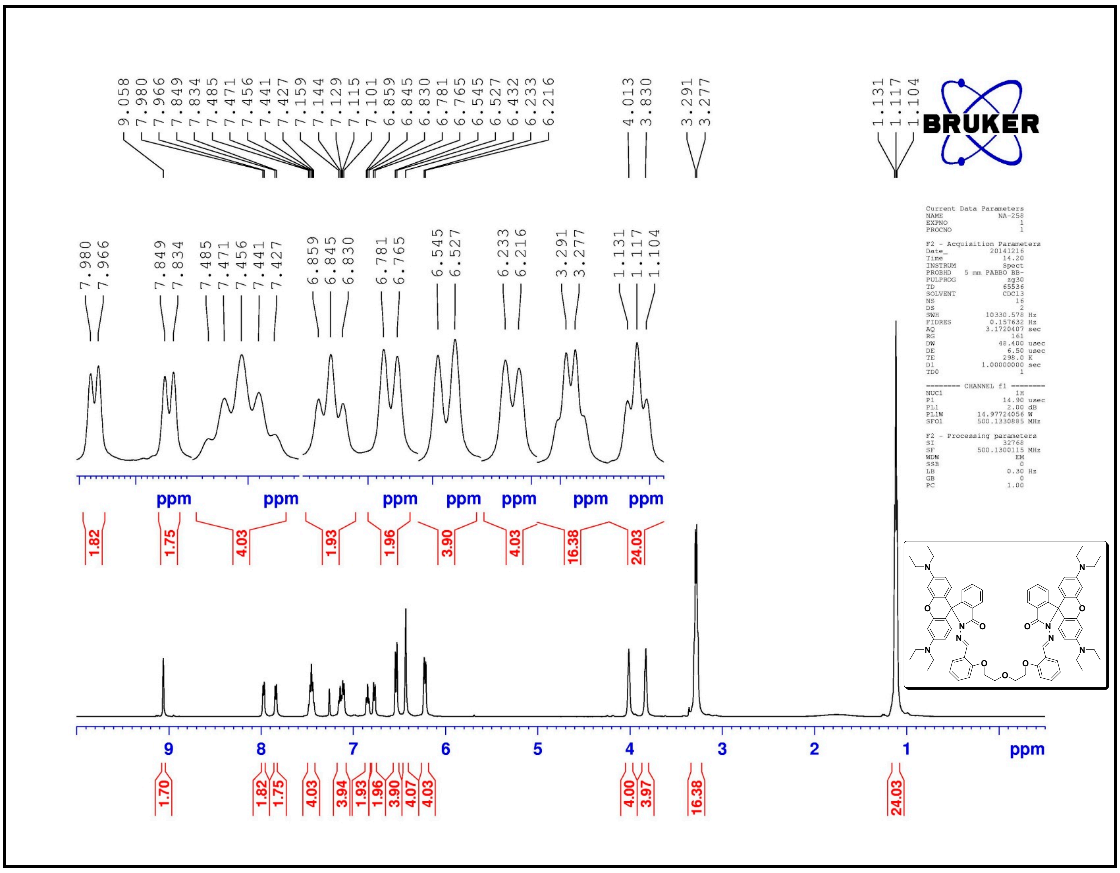

Figure S17. ^{1}H NMR Spectrum (500 MHz, $CDCl_3$) of **L3**.

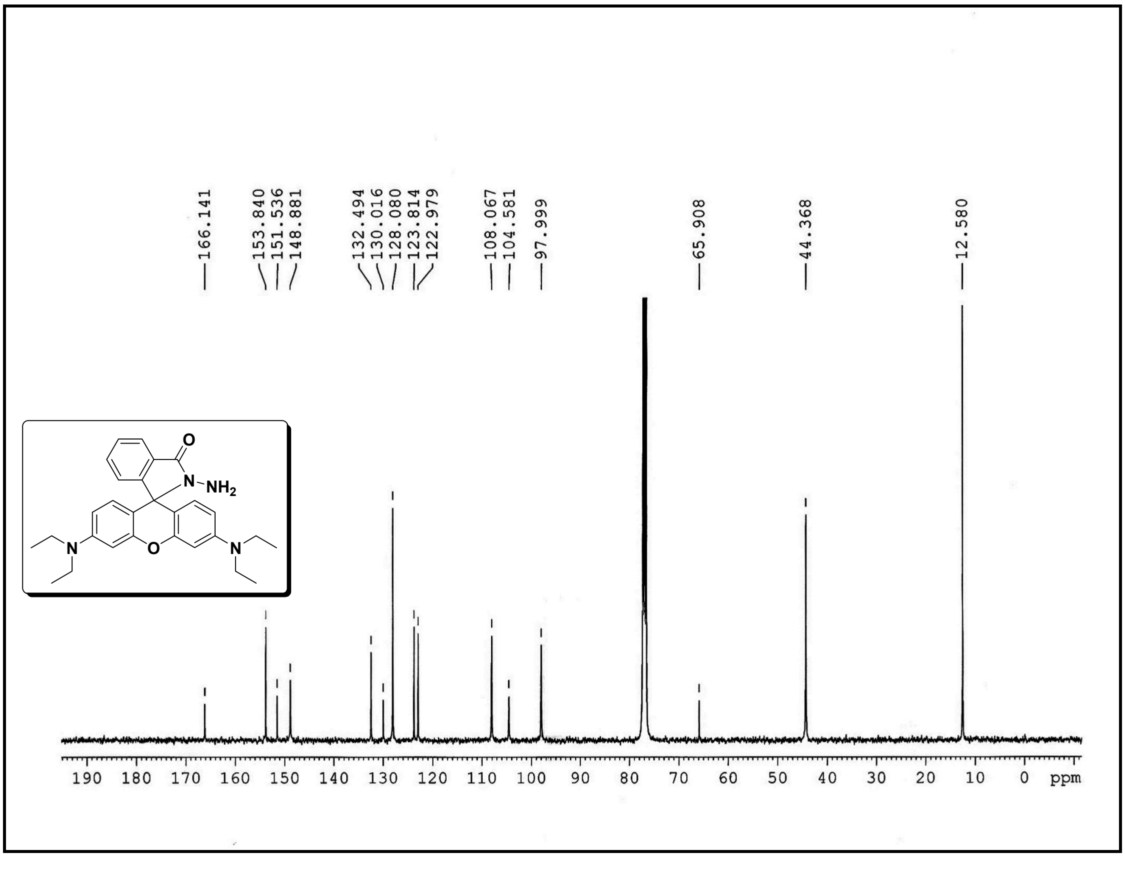

Figure S18. ^{13}C NMR Spectrum (500 MHz, $CDCl_3$) of **1**.

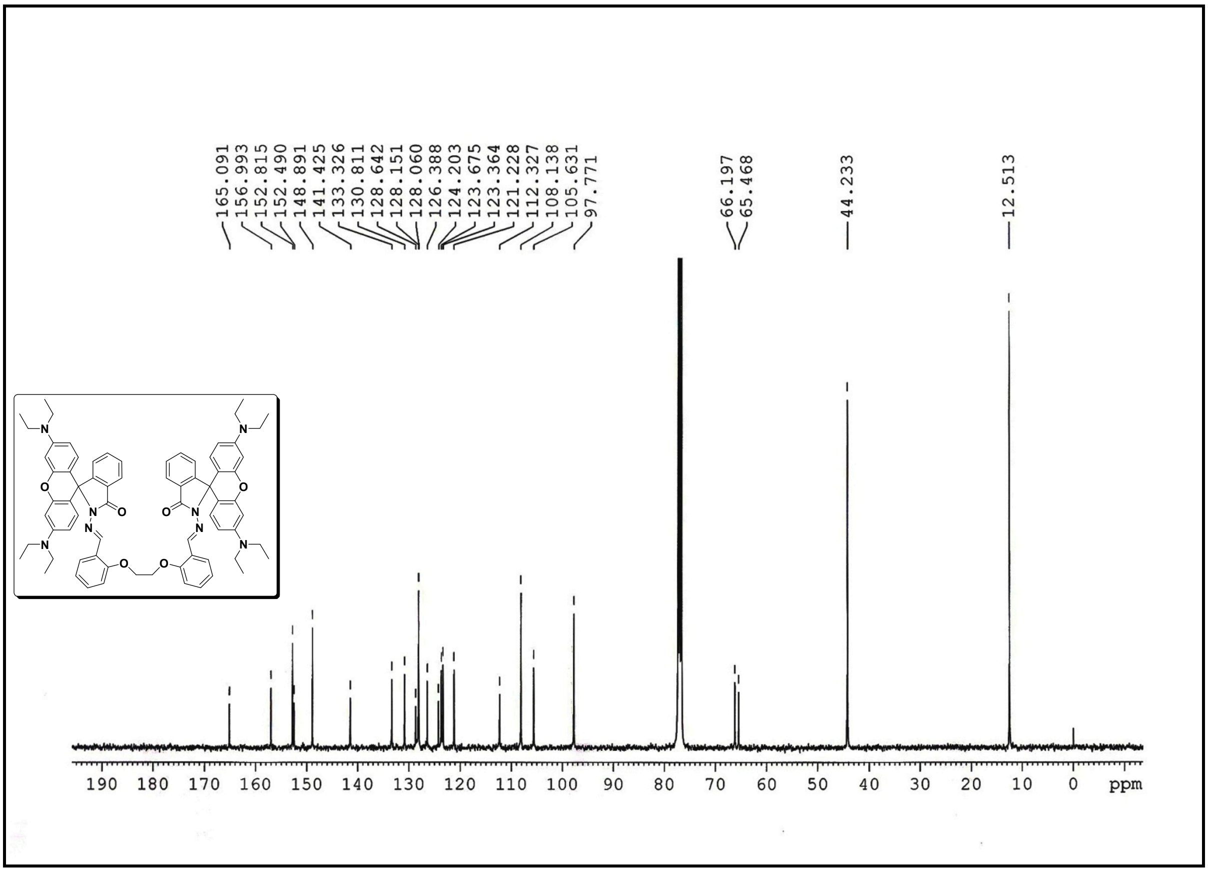

Figure S19. ^{13}C NMR Spectrum (500 MHz, $CDCl_3$) of **L1**.

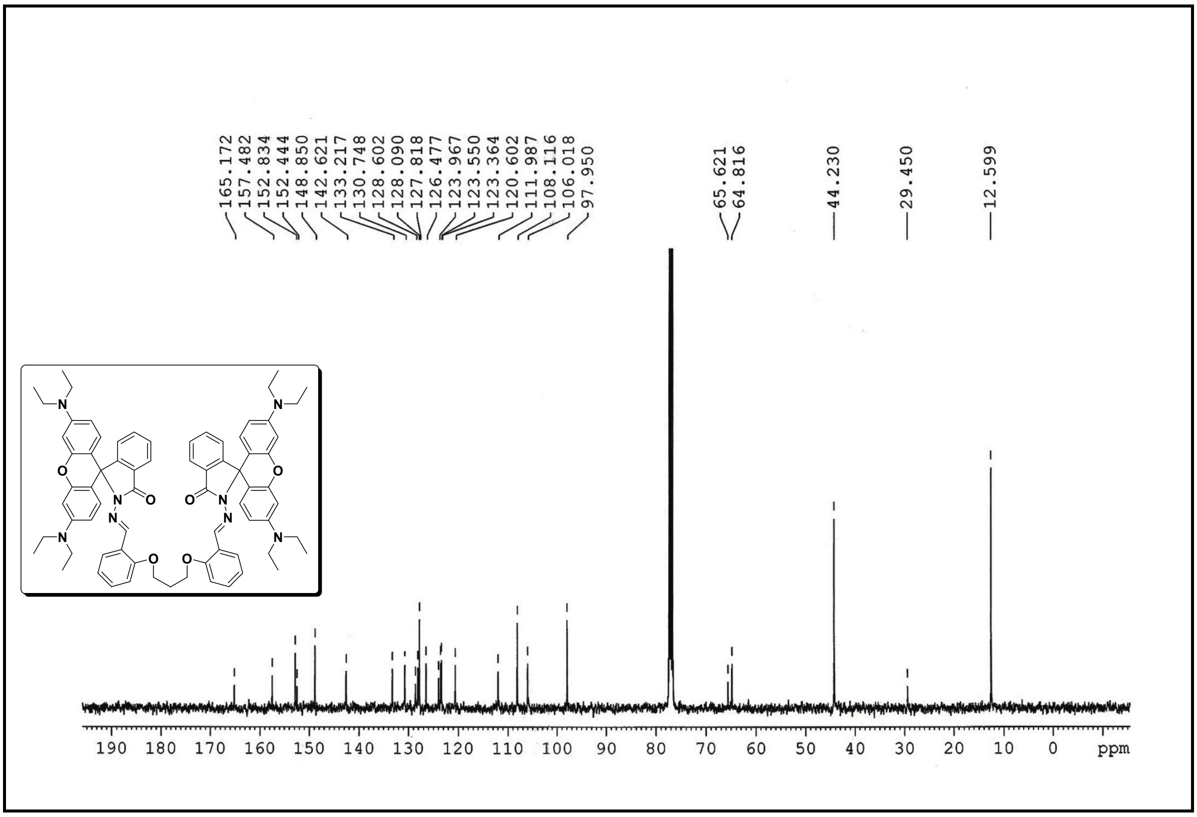

Figure S20. ^{13}C NMR Spectrum (500 MHz, $CDCl_3$) of **L2**.

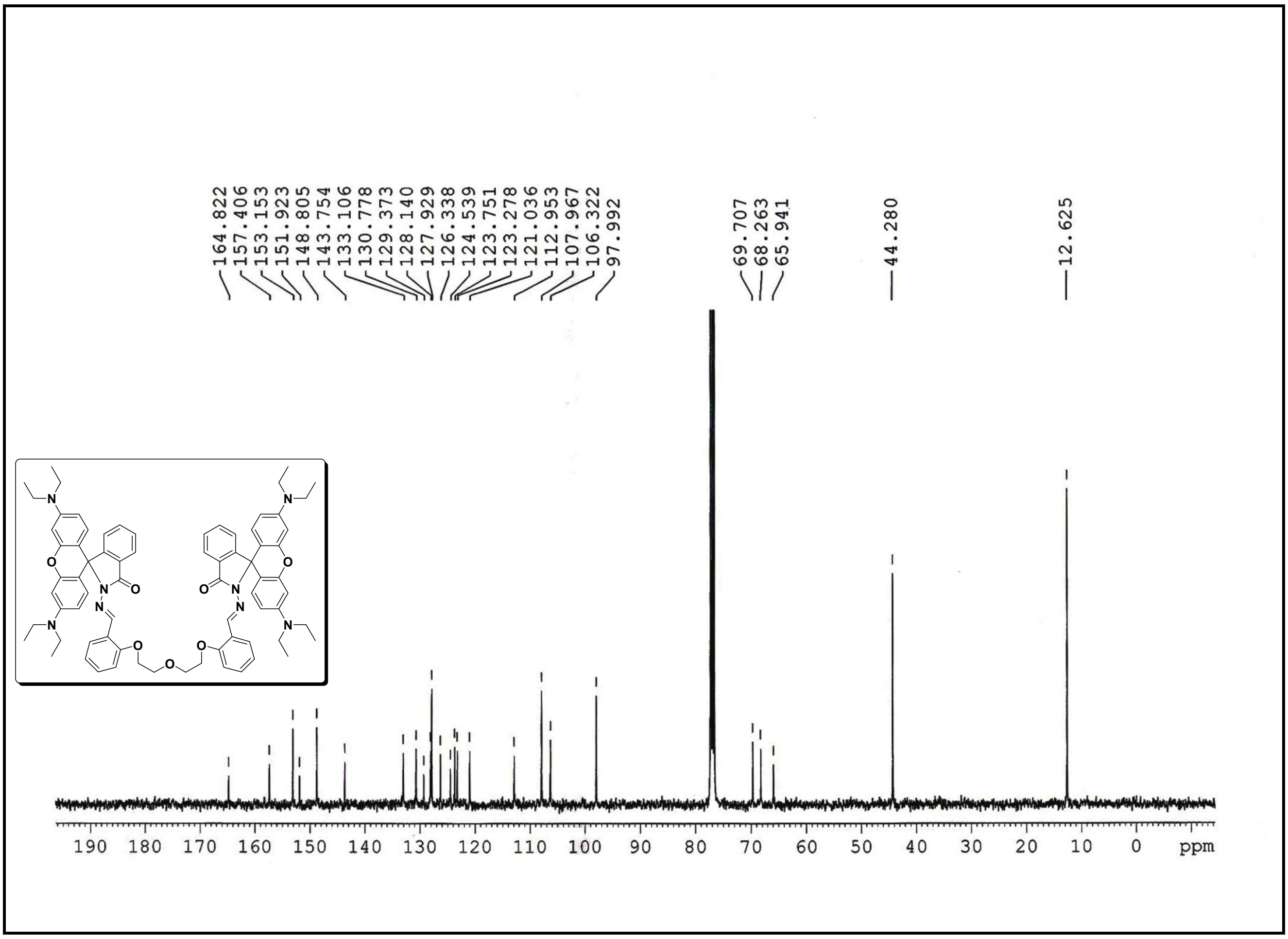

Figure S21. ^{13}C NMR Spectrum (500 MHz, $CDCl_3$) of **L3**.

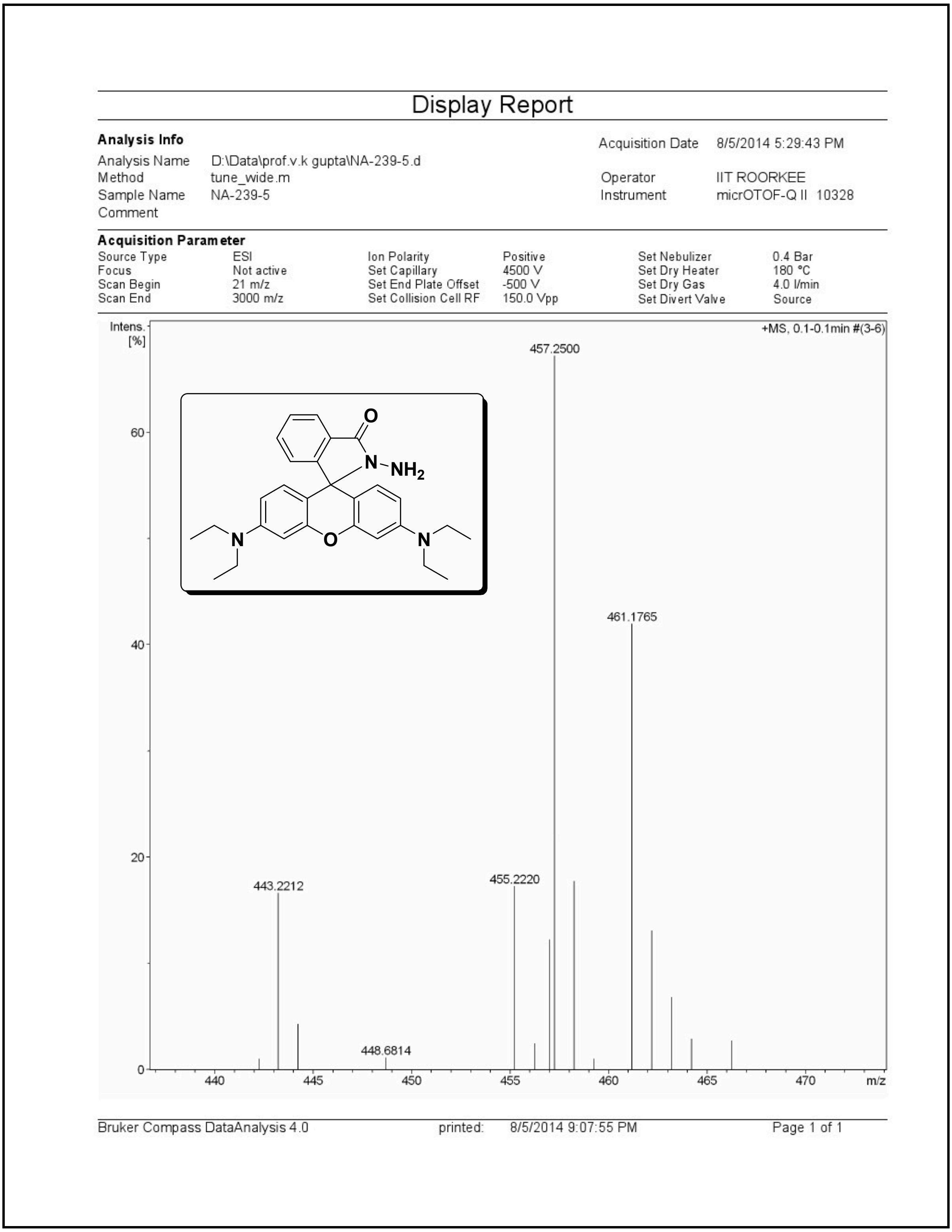

Figure S22. ESI-MS Spectrum of **1**.

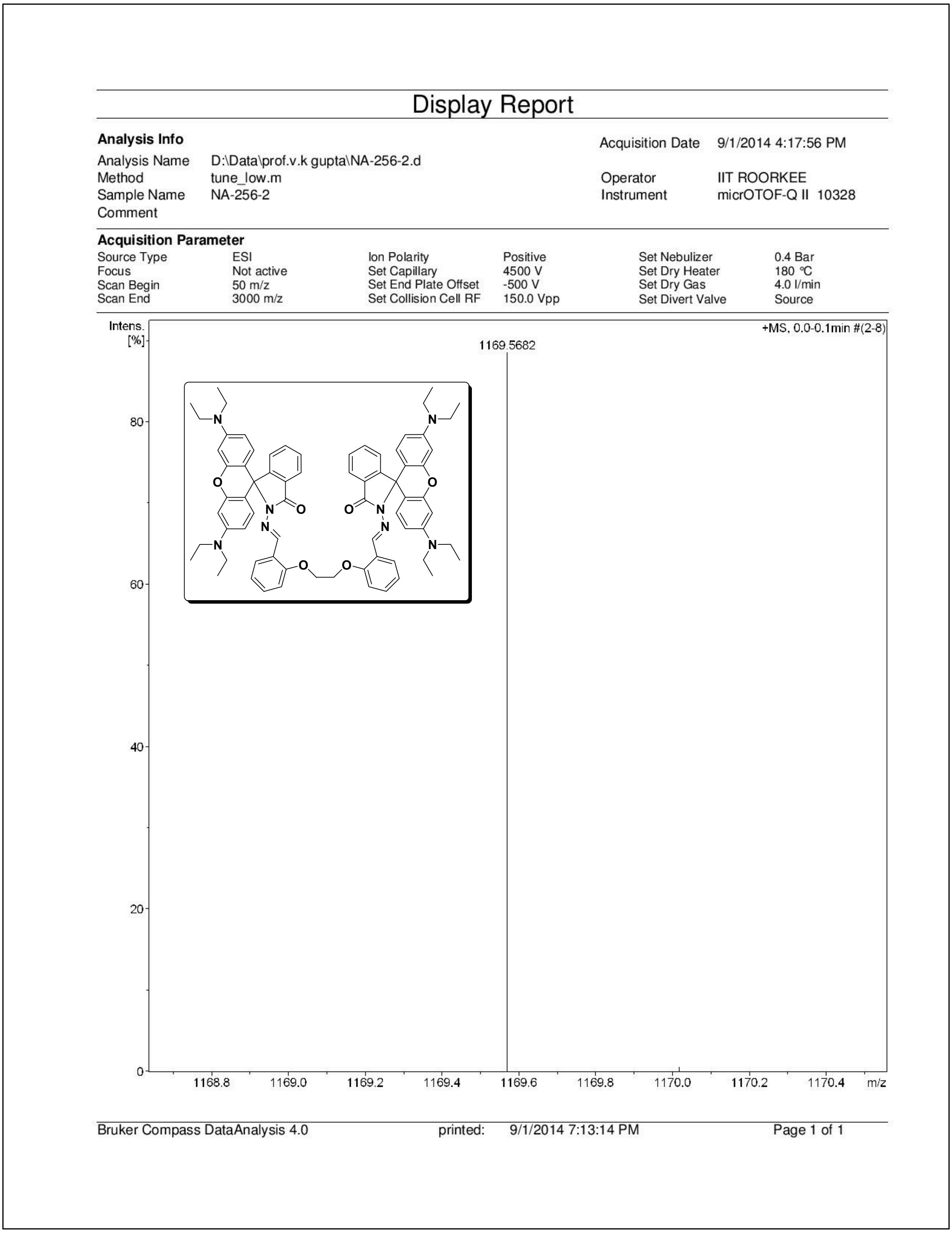

Figure S23. ESI-MS Spectrum of **L1**.

Display Report

Analysis Info

Analysis Name	D:\Data\prof.v.k gupta\NA-256-1.d	Acquisition Date	9/1/2014 4:15:50 PM
Method	tune_low.m	Operator	IIT ROORKEE
Sample Name	NA-256-1	Instrument	micrOTOF-Q II 10328
Comment			

Acquisition Parameter

Source Type	ESI	Ion Polarity	Positive	Set Nebulizer	0.4 Bar
Focus	Not active	Set Capillary	4500 V	Set Dry Heater	180 °C
Scan Begin	50 m/z	Set End Plate Offset	-500 V	Set Dry Gas	4.0 l/min
Scan End	3000 m/z	Set Collision Cell RF	150.0 Vpp	Set Divert Valve	Source

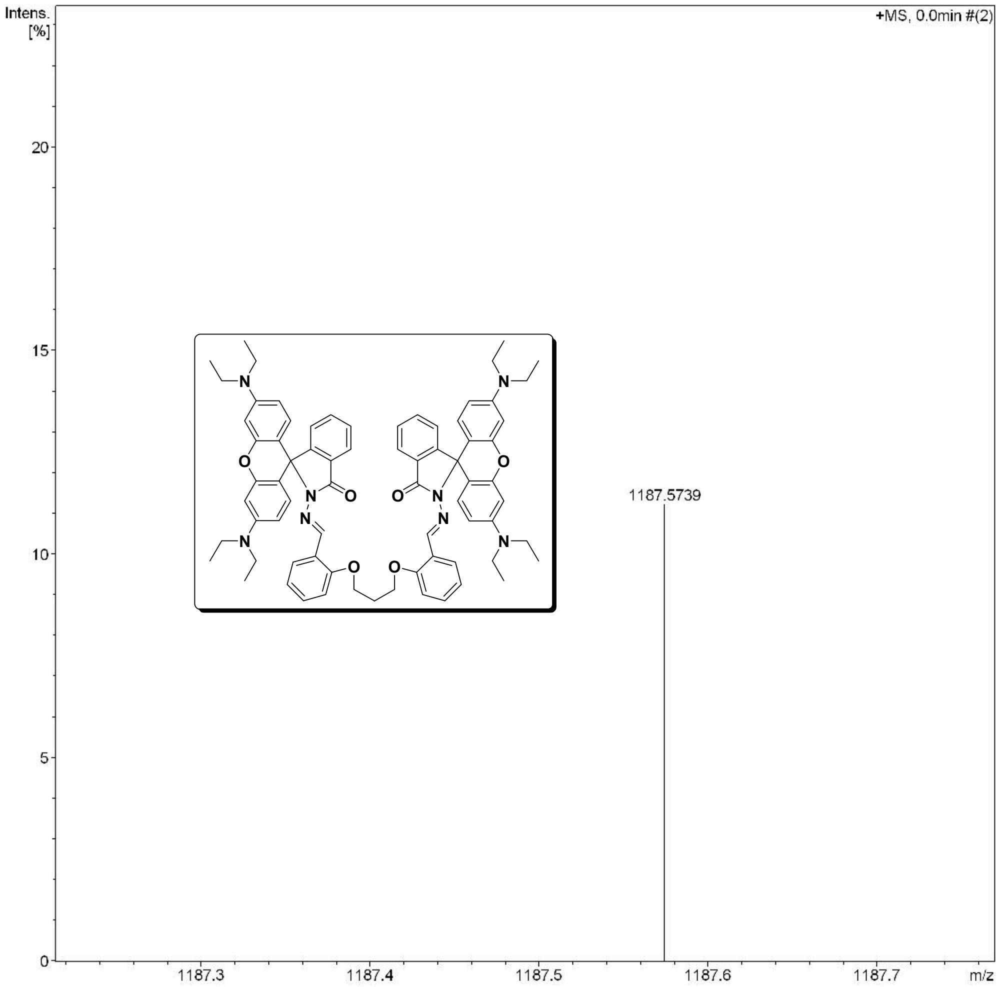

Bruker Compass DataAnalysis 4.0 printed: 12/29/2014 4:15:20 PM Page 1 of 1

Figure S24. ESI-MS Spectrum of **L2**.

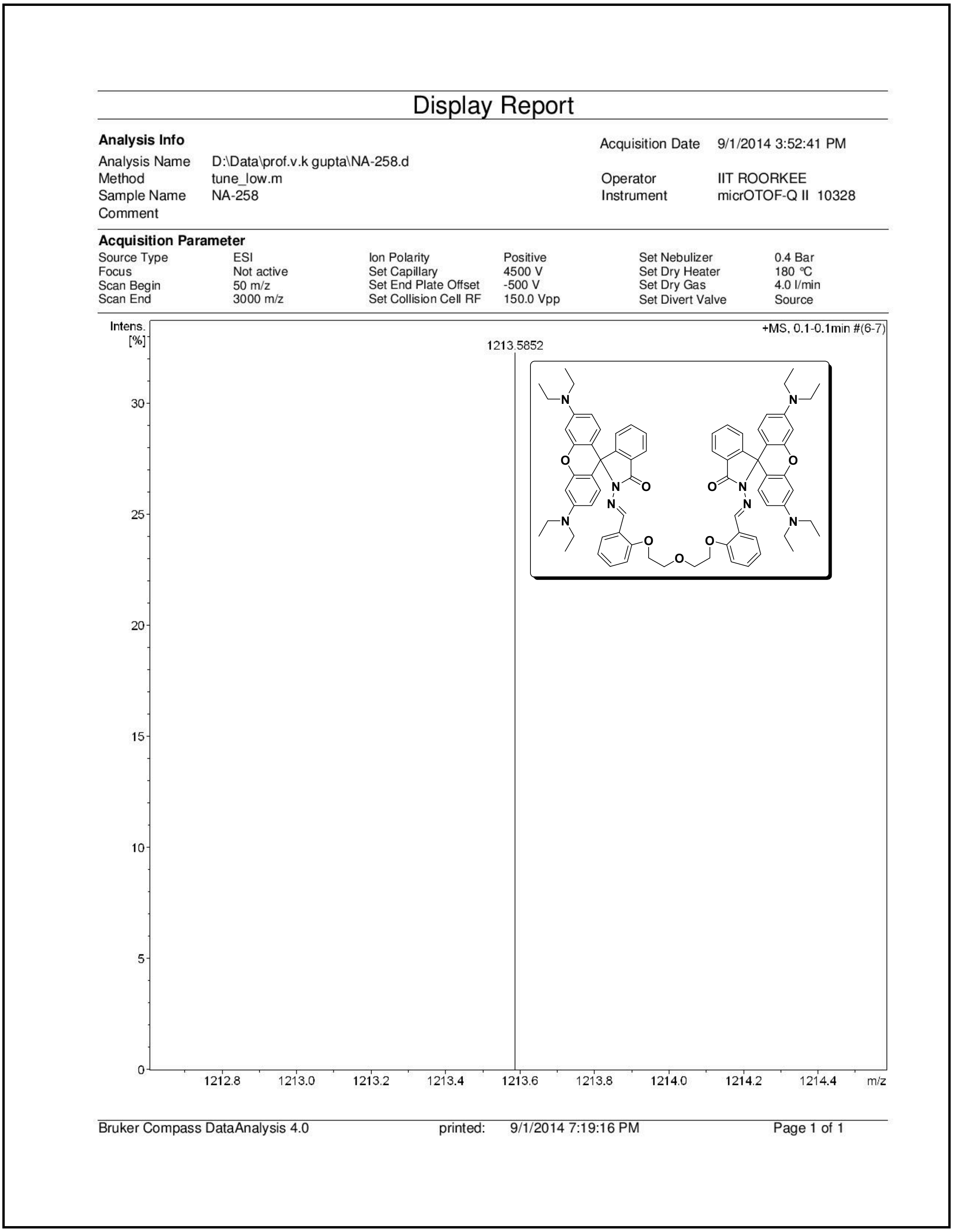

Figure S25. ESI-MS Spectrum of **L3**.

© 2015 by the authors; licensee MDPI, Basel, Switzerland. This article is an open access article distributed under the terms and conditions of the Creative Commons Attribution license (http://creativecommons.org/licenses/by/4.0/).
